# Human interpretable structure-property relationships in chemistry using explainable machine learning and large language models

**DOI:** 10.1038/s42004-024-01393-y

**Published:** 2025-01-14

**Authors:** Geemi P. Wellawatte, Philippe Schwaller

**Affiliations:** 1 Laboratory of Artificial Chemical Intelligence, Institute of Chemical Sciences and Engineering, Ecole Polytechnique Fédérale de Lausanne (EPFL), Lausanne, Switzerland; 2 National Centre of Competence in Research (NCCR) Catalysis, Ecole Polytechnique Fédérale de Lausanne (EPFL), Lausanne, Switzerland

**Keywords:** Chemical engineering, Cheminformatics, Computational chemistry

## Abstract

Explainable Artificial Intelligence (XAI) is an emerging field in AI that aims to address the opaque nature of machine learning models. Furthermore, it has been shown that XAI can be used to extract input-output relationships, making them a useful tool in chemistry to understand structure-property relationships. However, one of the main limitations of XAI methods is that they are developed for technically oriented users. We propose the XpertAI framework that integrates XAI methods with large language models (LLMs) accessing scientific literature to generate accessible natural language explanations of raw chemical data automatically. We conducted 5 case studies to evaluate the performance of XpertAI. Our results show that XpertAI combines the strengths of LLMs and XAI tools in generating specific, scientific, and interpretable explanations.

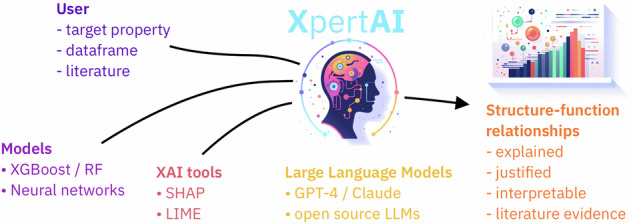

## Abstract (web summary)

Explainable artificial intelligence (XAI) can be used to extract input-output relationships, making them a useful tool in chemistry to understand structure-property relationships, but a key limitation of XAI methods is that they are developed for technically oriented users. Here, an XpertAI framework that integrates XAI methods with large language models is developed, enabling automatic generation of accessible natural language explanations of raw chemical data.

## Introduction

Understanding structure–property relationships has been a long-standing challenge in chemistry^1–3^ Seybold et al.^2^ highlight a fundamental concept in chemistry: the “properties and behaviors of molecules follow from their structures”. Therefore, elucidating these relationships facilitates the manipulation of molecules to achieve desired properties. Machine learning (ML) is a routinely used tool to complement human expertize, which solves complex tasks in chemistry by modeling structure–property relationships^4–8^. While ML has been proven to be successful in solving such tasks in chemistry^9–15^, experimental chemists often harbor skepticism toward predictions generated by such models, primarily due to the inherent opacity of these models. In essence, these ML models usually do not provide a rationale as to why a certain prediction was made. EXplainable Artificial Intelligence (XAI) is a new branch of AI that is rapidly growing and aims to explain the opacity nature of ML models. Therefore, developing XAI tools for chemistry is critical for increasing trust in ML models and expanding the possibilities of experimental and computational chemistry.

Justifications, explanations, and interpretability are three terms associated with XAI^16–18^. While a justification simply provides evidence for a prediction^19^, an explanation describes the rationale for the prediction^20^. However, the true potency of XAI lies in its interpretability, which concerns the extent to which a human can comprehend the provided explanation^16^. In a recent survey, Cambria et al.^21^ emphasized that there is a pressing need to refine the presentation of explanations. This means that although XAI addresses the opacity of ML models, they are not user-friendly for non-domain experts or non-technical users. Therefore, there is growing interest in incorporating natural language (NL) with XAI to produce more accessible explanations^21,22^. Furthermore, it’s worth noting that existing XAI methods often lack the flexibility to address specific user queries—can usually answer only one specific question, impeding their adaptability^23–26^. To meet this demand for creating intelligent, adaptable, and user-friendly XAI tools for chemistry, we introduce a Python package named “XpertAI”. Our tool combines XAI methods with large language models (LLMs) to extract structure–property relationships from raw data.

LLMs are generative models which can predict an output sequence given an input sequence. LLMs can be made into powerful agents that query databases, scrape and summarize literature, interpret, and generate text in NL^27^. Currently, there has been a surge in LLM-based research in chemistry and related sciences. For example, Zhiling et al.^28^ showed that ChatGPT could be used to accelerate text-mining and to predict metal–organic framework (MOF) synthesis using prompt engineering. Kan et al.^29^ showed that GPT-4^30^ language model can be used in parameter selection of polymer informatics. Furthermore, the authors highlight the importance of LLMs in research domains plagued with data scarcity. Boiko et al.^31^ introduced Coscientist, an AI system based on natural language, to design, plan, and execute chemical experiments. However, LLMs in isolation can be limited in addressing domain-specific problems within the field of chemistry. To circumvent such challenges at the intersection of chemistry and LLMs, Jablonka et al.^32^ demonstrated that finetuning LLMs could provide a solution to this. Relatedly, DARWIN^33^ is a series of finetuned open-source LLMs tailored for natural sciences. PMC-LLaMA^34^, Galactica^35^, and Med-PaLM^36^ are a few more examples of finetuned LLMs for scientific research. Following a different approach, Bran et al.^37^ showed LLMs can be enhanced to tackle tasks such as organic synthesis, drug discovery, and materials design by integrating external tools rather than finetuning.

### Motivation

Previously, it has been suggested that “black-box modeling first, followed by XAI” as a means to establish structure–property relationships without compromising accuracy or interpretability^18^. In this work, we present XpertAI, a framework that aims to establish connections between black-box models, XAI tools, and literature through LLMs to uncover relationships between molecular features and target properties. In a previous study, Seshadri et al.^38^ showed that LLMs combined with XAI can generate human-interpretable explanations. Unlike our approach, this work used LLMs only to summarize the findings from the XAI analysis in natural language. However, we show that LLMs combined with XAI tools and literature evidence, play a powerful role in generating both interpretable and scientifically accurate explanations. This work demonstrates that combining XAI with LLMs can be effectively used for hypothesis generation, marking a pioneering effort in this direction.

**Fig. 1:**
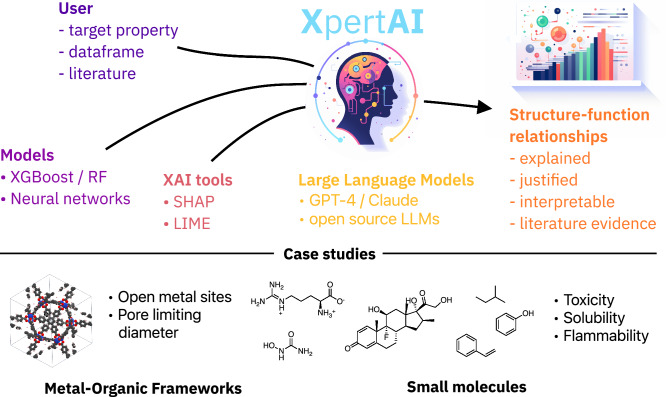
Overview of XpertAI. This tool combines XAI with LLMs to uncover human-interpretable structure–property relationships from raw data.

**Fig. 2:**
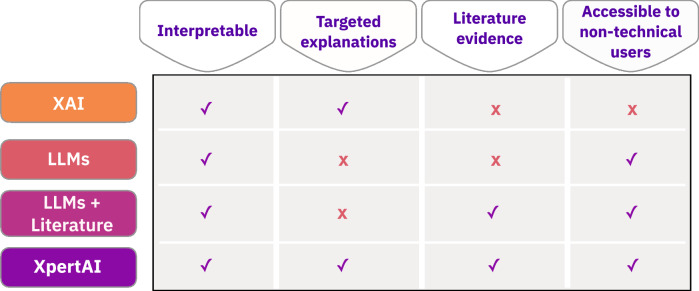
Attributes of XpertAI explanations. XpertAI explanations against baseline methods (XAI, LLMs, and LLMs + Literature) across four key attributes: interpretability, targeted explanations, incorporation of literature evidence, and accessibility to non-technical users.

As shown in Fig. 1, given a raw dataset, XpertAI employs XAI methods to identify crucial structural features that are correlated with the target property. Next, it draws on scientific evidence from literature to articulate structure–property connections based on these features. One key advantage of XpertAI is its ability to deliver precise natural language explanations (NLEs) tailored to specific datasets, as opposed to providing generalized explanations drawn from the broader literature. As illustrated in Fig. 2 XpertAI combines the strengths of XAI and LLMs in terms of specificity (to given data), interpretability, accessibility, and scientific nature of the explanations. In other words, XAI only directs the users to a trained model’s rationale and does not provide scientific reasoning, although the explanations may be interpretable. We show that LLMs can be used to address this limitation, thereby mimicking the practices that a scientist will follow to establish a hypothesis given raw data. To the best of our knowledge, currently, there is no such tool in chemistry that extracts NL structure–property relationships from user-given raw data. Furthermore, our application is generalizable to any domain that requires extracting input–output relationships as NLEs.

## Methods

We begin the workflow by training an ML model using the initial raw data. This model serves as a surrogate for mapping input to output. The initial data frame includes feature molecular structures and target labels for training. Note that these features must be human-interpretable (e.g., molecular descriptors/properties, and MACCS keys). However, XpertAI will also improve feature readability by default. Currently, we employ gradient-boosting decision trees with the XGBoost framework, utilizing the Scikit-learn API for regression and classification tasks^39,40^. We selected XGBoost as our default surrogate model because it has been shown to outperform many general neural network architectures regardless of its simplicity^41^. Additionally, this choice is motivated by training and inference efficiency, comparably higher interpretability, and ease of integration with XAI methods. Once the model is trained, users can select from SHAP^42^, LIME^43^, or both to estimate the “most impactful” features correlated with the molecular properties.

SHAP and LIME are possibly the most commonly used XAI methods to generate local explanations. They have been used in previous studies to interpret relevant features that contribute most towards the target properties in chemistry and adjacent domains^38,44–46^ In this study, we compute the mean SHAP values and *Z*-scores for LIME explanations to extract globally impactful features rather than generating local explanations. For the LIME analysis, we only use a sample of the initial dataset due to time and resource constraints. The default sample size is either 500 or the entire dataset if its length is less than 500. After identifying impactful features, we draw knowledge from the literature to elucidate physicochemical relationships between these features and the target property.

As seen in the overview of our proposed workflow (Fig. 1) LLMs are used to unite the backend modules generating human-interpretable explanations. More technically, XpertAI makes use of the retrieval augmented generation (RAG)^47^ approach to reliably generate scientific explanations using evidence gathered from the literature. LLMs’ inherent knowledge can be limiting in knowledge-intensive, data-sparse fields such as chemistry and materials science^48^. Therefore, LLMs are prone to generate misinformation and hallucinate answers in such cases. The RAG approach is commonly used to avoid such limitations in LLMs as it augments the LLM’s internal knowledge with external data sources^49^. It fundamentally consists of a retriever and a generator (the LLM). Usually, given a query, relevant chunks of text are retrieved based on a distance metric. We leverage on LangChain python package (https://github.com/langchain-ai/langchain) Chroma vector database (https://github.com/chroma-core/chroma) (the retriever) and, OpenAI’s GPT-4o^30^ language model (the generator) in this workflow. This GPT-4o version (gpt-4o-2024-08-06 at the time of publication) was trained on data up to October 2023 (https://platform.openai.com/docs/models/gpt-4o). Users have the flexibility to provide a literature dataset or scrape arxiv.org to gather relevant literature information. The latter is enabled via the arXiv python API.

We used a similar approach as the “StuffDocumentsChain” in LangChain to refine the explanations. First, we select the most related literature excerpts using maximal marginal relevance search (MMR). Then, the text excerpts are “stuffed” to a specialized prompt to generate the final explanation. We utilize the chain-of-thought prompting approach^50^ where a series of intermediate steps and examples are provided in the prompt to improve the output’s interpretability. Prompts are given in Appendix B. XpertAI also generates and adds citations in the final NLEs to improve the accountability of the explanations. We would like to highlight that, in addition to the NLEs, XpertAI also provides the surrogate model’s evaluation plot (error plot) and XAI analysis plots for the users. To streamline this complete workflow, we have deployed a Streamlit App (https://xpert-ai.streamlit.app/) that can be used with an OpenAI API key. More technically oriented users can implement XpertAI locally using our GitHub repository: https://github.com/geemi725/XpertAI.

## Results

We used the XpertAI tool to suggest structure–property relationships for five case studies in chemistry: (1) the presence of open metal sites in metal–organic frameworks (classification), (2) pore-limiting diameter in metal–organic frameworks (regression), (3) toxicity of small molecules (classification), (4) solubility of small molecules (regression), and (5) upper flammability limit of organic molecules (regression). Please note that we used the SHAP method as the chosen XAI method and its default hyperparameters to generate NLEs in the following case studies. We chose the SHAP method due to its consistency in generating global explanations in comparison to LIME. Complete NLEs and SHAP plots for each case study from XpertAI are provided in Appendices A and E in SI respectively. A set of published articles was uploaded for each case study to draw scientific evidence. These articles were manually curated based on relevance, number of citations, and the impact factor of the published journal. However, the relevance of the article to the task at hand was prioritized over other criteria. We only included peer-reviewed articles in this step to ensure scientific accountability. An additional technical benefit is the lower recall within the RAG framework. The references to the articles used in this work can be found in the XpertAI GitHub repository. Please note that XpertAI provides the option to automatically scrape articles from arxiv.org in place or in addition to uploading user-preferred literature.

### Case studies 1 and 2: structure–property relationships in metal–organic frameworks (MOFs)

MOFs, a hybrid class of materials in chemistry, consist of metal nodes connected by organic linkers^51^. Their porous nature lends them versatile properties such as gas separation and storage^52–54^, catalysis^55,56^, and drug delivery^57,58^. Understanding MOF structure–property relationships is crucial for optimizing their design in specific applications. Open metal sites, characterized by coordinative unsaturation, find valuable use in catalysis^51^. Additionally, the pore-limiting diameter is a key feature for screening them in selective gas capture applications^59^. However, the precise relationship between MOF atomic structure and open metal sites or pore-limiting diameter remains incompletely understood.

In case study 1, we sampled 4000 MOFs from the CoRE MOF 2019 database^60^ that contained labels for the presence of open metal sites and pore-limiting diameter. After input validation and the featurization step, we ended up with 3734 structures. These crystal structures obtained as CIF files were then featured using the CrystalFeatures tool^61^. Generated features are interpretable descriptors encompassing atomic and crystal characteristics, geometry features, and one-shot ab initio descriptors. Next, we uploaded the featured inputs and (binary) target labels as a CSV data frame along with a pre-selected literature dataset containing 41 publications to our XpertAI Streamlit App. The list of publications can be found in our GitHub repository. The generated NLE from XpertAI explains how (a) metals fraction, (b) density of solid, and (c) average cationic radius correlate with the presence of open metal sites. The XpertAI explanation aligns with the findings of Hall et al.^51^, where the authors identify metal identity and oxidation state, defect density, and site proximity as impactful structural components. Note that we omitted this review paper^51^ in the literature dataset uploaded to XpertAI to avoid data leakage.

Following a similar approach in case study 2, we used the same MOF dataset but with pore-limiting diameters as the label. Unlike case study 1, this is a regression-type problem. We uploaded a literature dataset with 24 journal articles to support XpertAI explanations. The pore size characterized by the pore-limiting diameter is an important property in MOFs that can control charge transfer and direct air capture^62^. According to XpertAI, key factors influencing the pore-limiting diameter include volume per atom, symmetry function G, and unoccupied energy levels at the conduction band. For instance, XpertAI hypothesizes that “Symmetry Function G1 may impact the pore-limiting diameter by influencing the spatial arrangement of atoms, potentially affecting the uniformity and size of the pores”. It continues to state that “an explicit relationship between Symmetry Function G1 and the pore-limiting diameter was not found in the given documents. However, the documents discuss the geometric properties of MOFs, which are inherently related to symmetry^63^”. This highlights XpertAI’s capability to produce insightful and plausible explanations while maintaining scientific rigor, avoiding speculative conclusions when supporting evidence is absent. We prompted XpertAI to go beyond merely identifying the most relevant molecular features associated with the target property. It also hypothesizes potential structure–property relationships and offers scientific reasoning, drawing on insights from the provided literature, emulating the approach a human scientist would take. For a comprehensive textual explanation, please refer to the Supporting Information (Appendix A).

### Case study 3: small molecule toxicity

Toxicity prediction of small molecules is a benchmark task in chemistry, particularly in drug discovery^64,65^. Despite the extensive research in this area, a precise understanding of the relationship between molecular structure and toxicity remains elusive. In this case study, we sampled and validated 1478 molecules from the Tox21 database^64^ where binary labels indicate toxicity (a classification task). Then we featurized the input molecules in SMILES format using MACCS descriptors^65^ implemented in the RDKit package^66^. These descriptors are human-interpretable binary features containing 167 yes/no questions regarding molecular structure. Additionally, we used 45 manually curated journal articles to gather scientific evidence for the XAI observations. XpertAI identifies the presence of a heteroatom bonded to three oxygen atoms, tertiary amine and carbon-oxygen single bond to be associated with the toxicity of molecules. The generated XpertAI explanation summarizes: “The analysis of features identified by XAI in relation to the toxicity of small molecules reveals several key insights. The presence of a heteroatom bonded to three oxygen atoms, such as in phosphate groups, is associated with increased chemical reactivity due to the electronegative nature of oxygen atoms, potentially influencing toxicity. Tertiary amines, known for their nucleophilic properties, may interact with electrophilic sites in biological systems, contributing to toxicity. Additionally, carbon-oxygen single bonds, prevalent in functional groups like alcohols and ethers, can affect the solubility and reactivity of molecules, thereby impacting their toxicity. These features highlight the complex interplay between chemical structure and biological activity, underscoring the importance of understanding molecular interactions in toxicity predictions.” We note that the XpertAI explanation aligns with the findings in work by Meanwell^67^ and Limban et al.^68^ which state that aromatic amines and nitro groups are associated with increasing molecular toxicity. These references were not included in the literature dataset uploaded to XpertAI. The complete XpertAI NLE can be found in SI (Appendix A). While XpertAI suggests that these features can alter toxicity as they affect the reactivity of the molecules and their ability to form reactive species, it’s important to note that toxicity is a complex property that is likely influenced by a combination of many features.

### Case study 4: small molecule solubility

Aqueous solubility of small molecules is a critical property in drug discovery as solubility determines the interaction of the drug in a biological environment^69^. To explain the relationship between the molecular structure and its solubility, we used a sample dataset with 9982 molecules from the AqSolDB^70^ dataset for training. Once again, we used MACCS descriptors to convert the molecules into a binary vector.

**Fig. 3:**
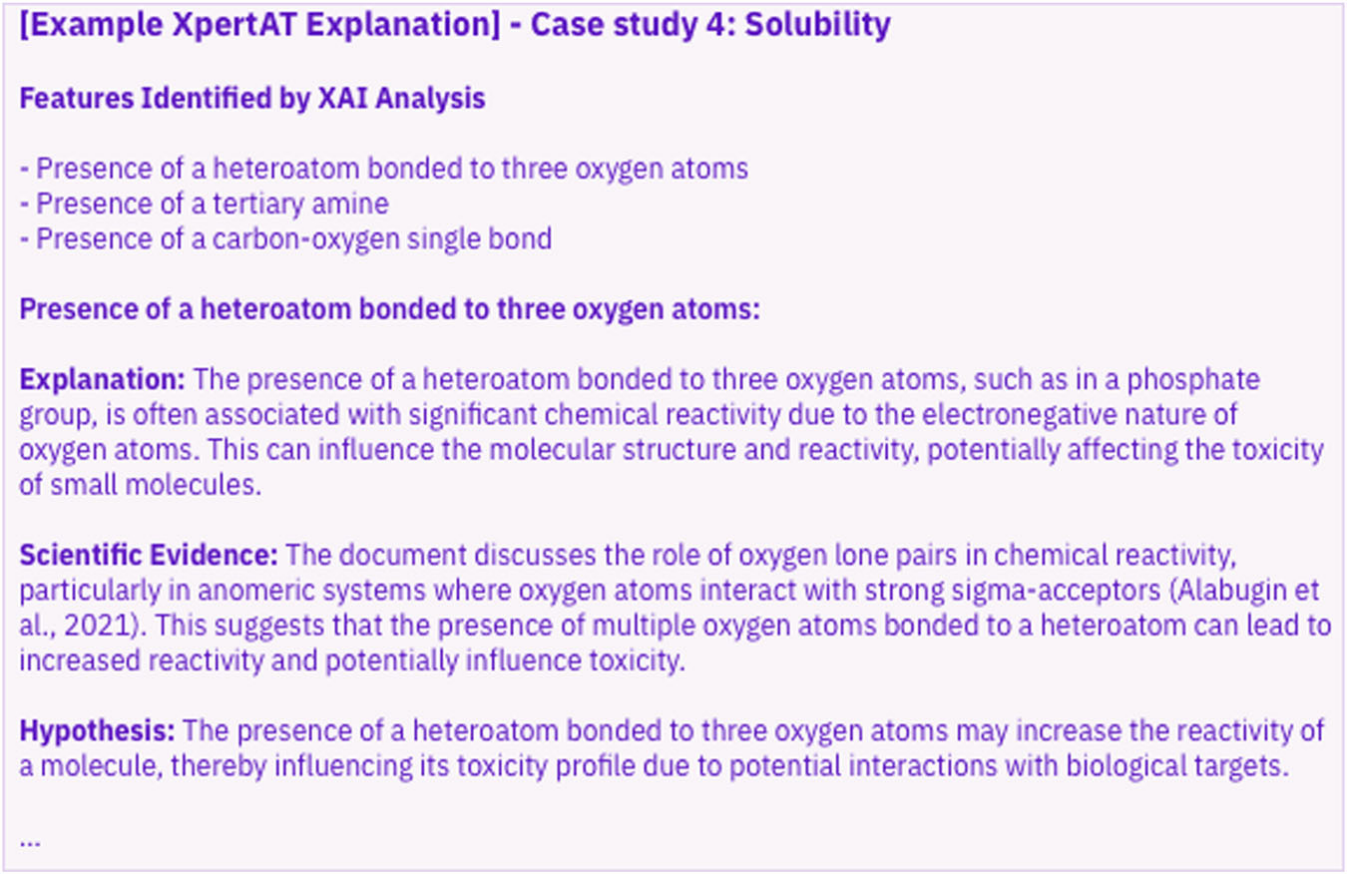
An excerpt from the XpertAI exaplnation. This illustrates how XpertAI combines features identified through XAI analysis, scientific evidence, and hypotheses to provide interpretable and targeted insights into molecular behavior.

We uploaded a literature dataset with 27 related publications. References to these can be found in our GitHub repository. XpertAI explains the structure–solubility relationship as follows. “The features identified by the XAI analysis, such as the presence of an atom at an aromatic/non-aromatic boundary, two heteroatoms bonded to each other, and an atom with three heteroatom neighbors, all show strong negative correlations with solubility. These features likely influence solubility by affecting molecular planarity, symmetry, electronic distribution, and hydrogen bonding potential. The presence of aromatic boundaries and heteroatom interactions can maintain molecular structures that are less favorable for solubility, as supported by the SHAP analysis and literature on molecular modifications for solubility enhancement (XpertAI, 2024)”^71,72^. Figure 3 is an additional expert from the XpertAI explanation with its native formatting. Complete explanations can be found in SI (Appendix A). This further demonstrates XpertAI’s accessibility as a tool that generates credible, natural language explanations of molecular structure–property relationships. To the best of our knowledge, XpertAI is currently the only tool that combines explainable AI (XAI) with large language models (LLMs) to interpret such relationships.

### Case study 5: compound flammability

The upper flammability limit (UFL) of an organic compound is an important characteristic that determines the hazardous nature of the compound^73^. This is an interesting topic of study in both experimental and computational studies^74–77^. We used the UFL dataset used by Yuan et al.^73^, which was extracted from reference Crowl and Louvar^78^. This dataset only contained 79 organic compounds. We used the same quantum and non-quantum molecular descriptor set used in ref. ^73^ to feature the molecules. After uploading the initial dataset and 15 curated publications, we obtained the following explanation. “The features identified by the XAI analysis, including the structural information content index (neighborhood symmetry of zero-order), information content index (neighborhood symmetry of order), and dipole moment, all play significant roles in determining the upper flammability limit (UFL) of organic molecules. The Structural Information Content Index (neighborhood symmetry of zero-order) and its generalized form, information content index (neighborhood symmetry of order), quantify molecular symmetry, which influences molecular stability and reactivity. These factors can be crucial in determining how easily a molecule can ignite and sustain combustion. The dipole moment, although not explicitly discussed in the provided documents, could affect molecular interactions and stability, thereby influencing flammability. These features collectively provide a comprehensive understanding of the molecular characteristics that impact the UFL of organic compounds.”

This case study is intentionally used as a negative example. We deliberately selected a smaller dataset with limited supporting literature, anticipating that the surrogate model would not be fully trained and may potentially produce spurious relationships. Furthermore, cross-referencing revealed that the provided literature did not explicitly identify any correlations between the features examined and the upper flammability limit (UFL). As provided in SI Appendix A, XpertAI provides a complete, textual explanation extracted from raw data. However, this may not correctly reflect the underlying molecular structure–property relationship.

## Evaluations

Firstly, to evaluate the explanations for the listed case studies, we compared three different explanations from: (1) XpertAI, (2) ChatGPT (GPT-4o), and (3) Graphical plots from the XAI analysis. The aim was to evaluate if XpertAI can leverage the advantages of both XAI and LLMs, rather than using one alone. We asked five expert chemists (graduate students in chemistry) to score 15 explanations in total (5 tasks × 3 explanations). The experts were given a scorecard (given in SI’s Appendix D) to evaluate each answer based on accuracy, interpretability, accessibility, usefulness in research, and specificity to given data. Each category was given an arbitrarily selected maximum score of 6 for evaluation purposes. Values per answer are given next to answers in Appendix D.

**Fig. 4:**
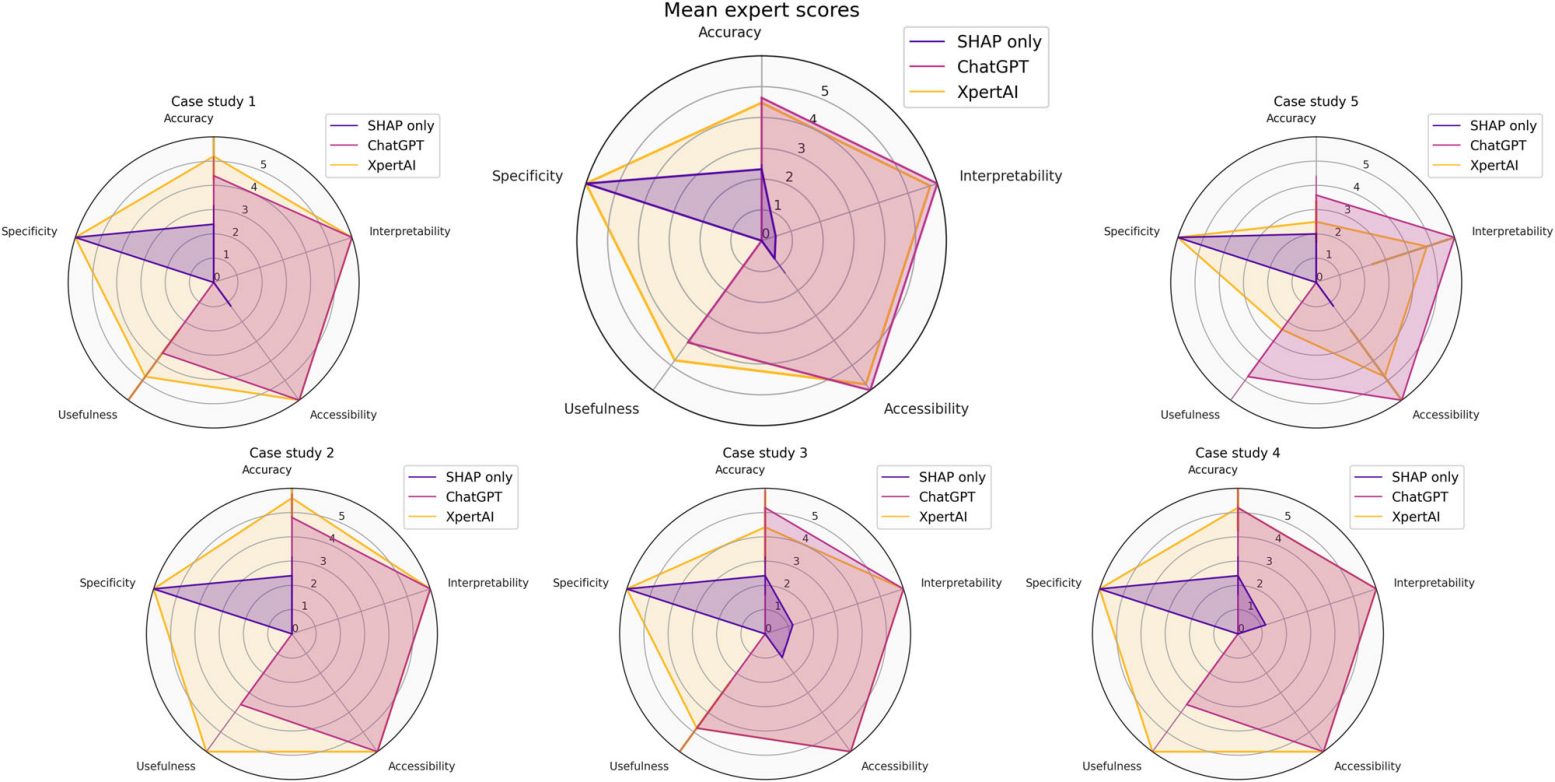
Human expert consensus for all case studies and the mean scores. Five human experts (graduate students in chemistry) were asked to evaluate explanations from XpertAI, ChatGPT, and SHAP plots for each case study based on accuracy, interpretability, accessibility, usefulness in research, and specificity to given data. Each category was given a maximum score of 6.

As seen in Fig. 4, on average, evaluators scored XpertAI NLEs highly under each category. In all 5 tasks, experts identified that ChatGPT explanations are not tailored to the given dataset. Contrastively, evaluators agreed that while XAI results (SHAP plots in this case) are specific to given data, these lack accessibility and interpretability. Often, XAI plots were labeled as not useful for further research. On the other hand, it can be inferred from Fig. 4 that XpertAI explanations were preferred in terms of interpretability, reliability, and specificity. As summarized in Fig. 2, the evaluations conclude that XpertAI effectively combines the advantages of both ChatGPT and XAI to provide a complete explanation. The expert scores further validate the accomplishment of our goal to extract accessible and interpretable structure–property relationships in chemistry from raw data. Based on expert scores for individual studies, we noted that for case studies 1–4 XpertAI’s explanations always scored better or as equally as ChatGPT, in terms of interpretability, accessibility, and usefulness. As expected, for our negative case study, experts scored XpertAI lower than ChatGPT in all categories except for specificity. We point out that, this is possibly due to inadequate training of the surrogate model used in the XAI study. We highlight that XpertAI’s success is dependent upon both the XAI method and LLM’s capabilities. A surrogate model that can capture the “True” relationship as closely as possible can accurately identify the most impactful molecular features, LLM’s performance determines the interpretability and credibility of the generated explanations. Additionally, it should be emphasized that the quality of the generated explanations is also dependent on the quality of the literature dataset provided. In all 5 case studies, we used manually curated, peer-reviewed journal articles only. These were selected based on the number of citations, relevance, and the impact factor of the journal. Note that a systematic selection of literature articles balances the precision and recall of the generated references. The lists of references used can be found in our GitHub repository.

Additionally, we observe that XpertAI scored comparatively less in case study 3. We argue this is because human experts excel at validating broad scientific understanding and not knowledge specific to a given dataset. However, XpertAI’s explanations are specific to relationships established from provided datasets, which may be different from familiar concepts in chemistry. While ChatGPT can generate broad explanations that can resemble general chemical understanding, these explanations are vulnerable to hallucinations and misinformation. The advantage of XpertAI is that it overcomes these vulnerabilities by using the RAG approach.

We further evaluated the precision of the generated hypotheses by XpertAI and compared with GPT-4o explanations. For each task and each feature that is identified in the XpertAI explanation, we extracted each proposed hypothesis and labeled the correlation of the feature with the property under study (OMS, PLD etc.) as positive, negative, or unclear. An example of an unclear correlation is “Metal ions with favorable redox properties might be more likely to form stable open metal sites.” We used the following formula to compute the precision.

1 $${{{\rm{precision}}}}= 	\frac{1}{{N}_{{{\rm{features}}}}\times {N}_{{{\rm{runs}}}}}{\sum}_{i}^{{N}_{{{\rm{features}}}}}\left\vert (1)\times {n}_{i,{{{\rm{positive}}}}}+(-1)\times {n}_{i,{{{\rm{negative}}}}} \right. \\ 	\left.+(0)\times {n}_{i,{{{\rm{unclear}}}}}\right\vert$$

**Fig. 5:**
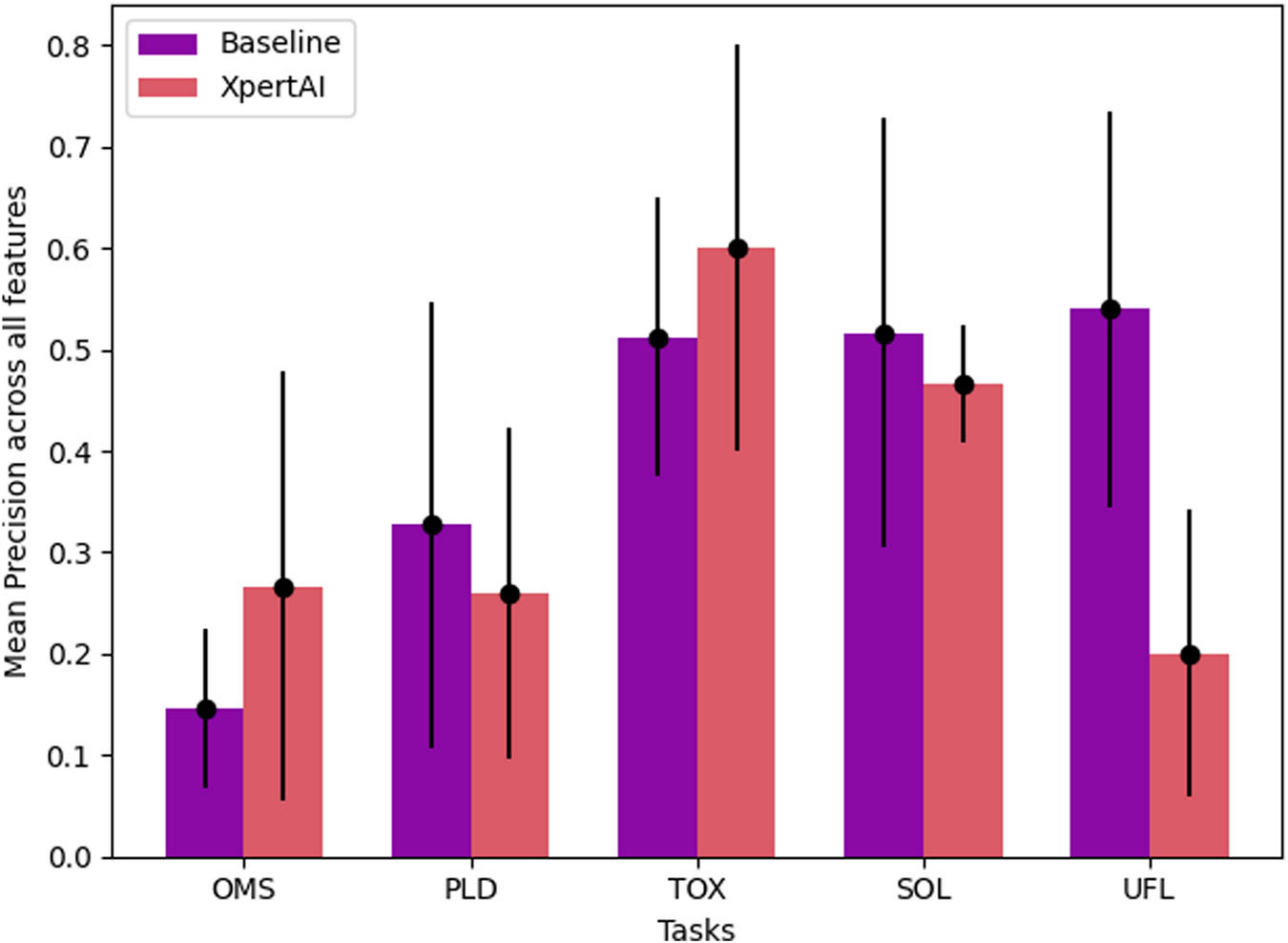
Mean precision of hypotheses generated (*↑*). XpertAI-generated hypotheses are compared with baseline GPT-4o. Equation (1) was used to compute the precision  ∈ {0, 1}. Error bars represent the standard deviation.

Here, *N*_features_ is the total number of unique features listed in the explanations, and *N*_runs_ = 5. assigned arbitrary weights of +1, −1, and 0 to calculate precision  ∈ 0, 1. These weights allow us to assess whether the generated explanations align or diverge at the feature level. The results shown in Fig. 5 reflect this analysis. XpertAI either outperforms or is comparable to the baseline at the feature level, except for case study 5, our negative example. In instances where XpertAI scores were lower, the majority of per-feature correlations were classified as unclear, reflecting the absence of explicit correlations in the literature. This suggests that XpertAI avoids generating speculative or unfounded conclusions. Conversely, while the GPT-4o baseline model demonstrated more consistency in its claims across the five runs, there is no assurance that these claims or correlations are free from hallucinations.

Next, to assess the overall content of XpertAI and ChatGPT explanations, we asked Claude AI assistant (https://claude.ai/) by Anthropic https://www.anthropic.com/ to compare the two explanations based on relevance, accuracy, and interpretability. The complete responses from Claude are given in SI (Appendix C). Based on the responses, Claude rates the explanations from XpertAI higher than ChatGPT’s for all case studies except for case study 5. We summarize the evaluations by Claude for ranking XpertAI’s explanations higher. Note that we anonymized the two explanations during the scoring; Explanation A is by XpertAI, and Explanation B is by ChatGPT. Each evaluation was run independently to avoid model biases.

- Explanation A directly discusses the specific features identified by the XAI analysis and provides concrete examples of how changing those features affects the target property, indicating high relevance for research. Explanation B provides a more general background on how molecular structure influences the target properties. While still relevant, it does not directly address the specific features called out in the XAI analysis.
- By extensively referencing multiple recent studies on the topic, Explanation A establishes accuracy in its explanations. The research evidence lends credibility and precision to the statements made. Explanation B does not provide any citations, making its accuracy more uncertain.
- Explanation A is more narrowly tailored to the specific question of how the features identified in the XAI analysis impact target properties. It provides mechanistic interpretations, examples, and literature references. This level of relevance, interpretability, and accuracy makes Explanation A better suited for guiding further research compared to the more general background provided in Explanation B.

However, for case study 5, Claude ranked the ChatGPT explanation higher. This is similar to the observations from expert evaluations. According to Claude, Explanation B provides a more thorough and relevant discussion of how molecular structure impacts the upper flammability limit. It covers key structural factors and gives useful insight into the research question. However, Explanation A while relevant, lacks the comprehensive coverage of Explanation B. We hypothesize this is due to the underperformance of the trained XGBoost model, as evidenced by a higher root-mean-square error (RMSE) during testing, which results in XpertAI’s lower-rated explanation. This possibly stems from the limited size of the training dataset (only 63 data points for training). As a result, the XAI analysis reveals the model could have learned non-causal correlations within the dataset. Therefore, the essential features may not be faithfully represented, thereby leading XpertAI to generate a flawed explanation.

Next, we validated the accuracy of the citations. Based on the results given in Table 1 we see that XpertAI’s citations are accurate and relevant. Although XpertAI incorrectly cites ref. ^60^ in case study 2:PLD, the text mentions the acronym PLD. However, in cases where XpertAI does not find explicit relationships in the text, it highlights this and avoids false citations.

**Table 1:** Quantitative analysis of citation accuracy in XpertAI explanations

Case study	Citation accuracy	Comments
OMS	2/3	Ref. ^60^ was neither correct nor relevant.
PLD	1/2	Ref. ^60^ mentions PLD, but the citation was incorrect.
TOX	1/1	No issues with citations.
SOL	2/2	No issues with citations.
UFL	2/2	No issues with citations.

Based on our analyses, we conclude that XpertAI’s overall cross-referencing performance is satisfactory. However, we observed that one of the main causes of failure stems from the absence of feature labels in the scientific text. For example, in case studies 2 and 4, XpertAI attempts to cross-reference feature label such as “symmetry function G1”, “unoccupied energy levels at conduction band minimum”, and “presence of an atom with three heteroatom neighbors” in the provided literature corpus. We therefore underscore the importance of carefully selecting feature descriptions. Although XpertAI can search for synonymous descriptors when the exact label is unavailable, this process can result in deviations from the original XAI findings. We note significant room for improvement within the XpertAI framework by increasing the number of provided literature articles, although this would lead to increased recall. Additionally, providing XpertAI access to automated literature scraping tools can significantly enhance its capacity for scientific explanation. This expanded toolkit allows the agent to perform more comprehensive analyses, utilize specialized resources, and deliver more accurate and nuanced interpretations of complex scientific concepts. There are numerous leading works investigating this specific problem, which are beyond the scope of this study.

## Open-XpertAI: exploring open-source LLMs

In the previous sections, we demonstrated that XAI combined with GPT-4o garners significant advantages in explaining structure–property relationships in molecules. However, a notable limitation of this approach lies in the proprietary nature of GPT-4o, necessitating a paid license for its utilization. Conversely, there exists a substantial array of open-source LLMs that have demonstrated remarkable prowess in text generation. In addition to the financial benefits, open-source LLMs also provide transparency, flexibility, and the benefit of community contributions. Nonetheless, it is important to acknowledge that despite these advantages, the overall performance of these models still trails behind that of GPT-4o. In a related study, Bai et al.^79^ evaluated the applications of open-source LLMs in MOF research.

To investigate the feasibility of integrating open-source LLMs into XpertAI, we conducted a brief study. We assessed the performance of 4 open-source LLMs that have demonstrated comparable capabilities to GPT-4, focusing on the accuracy of their generated explanations. The selected LLMs were: Llama3.1:8b^80^, Llama2:7b^81^, Mixtral:8 × 7b-instruct-v0.1-q5_0^82^, Starling-lm:7b-alpha^83^ and Phi:2.7b^84^. These open-source LLMs were configured and executed locally utilizing Ollama, a streamlined AI tool designed for the local deployment of open-source LLMs. For all LLMs except Mixtral:8 × 7b-instruct, Q4_0 quantization level was used. While we utilized a selection of open-source LLMs available at the time of research, we acknowledge that more advanced models may be available at the time of publication, and future studies could benefit from these enhanced versions. Detailed performance metrics of each LLM against benchmark datasets can be found in the respective references.

This study aims to assess the ability of open-source LLMs to accurately generate explanations within an RAG system like XpertAI as an alternative to proprietary LLMs. We generated explanations from these LLMs using the same molecular features and literature data employed in previous case studies. Then a human evaluator was asked to assess the accuracy of the generated explanations for all 5 case studies. The evaluator assigned a score of 1 for accurate explanations and 0 for inaccurate ones. The scores for references were averaged by the number of references in each explanation before totaling. Please see Table 2 for the summarized results. We generated 5 explanations per case study and computed RougeL scores to gauge the variation among explanations. The average RougeL scores over the 5 case studies are given in Table 2. Although the RougeL score can be used as a measure of variability in the content, this is not an indication of the accuracy of the explanations. Please see SI (Appendix F) for RougeL scores for individual case studies. In our analysis, we noted that some LLMs have higher RougeL scores than XpertAI explanations. However, XpertAI outperformed all open-source models across the listed metrics in Table 2. From the results, we observe that mixtral (8 × 7b-instruct-v0.1-q5_0), Llama3.1:8b, and starling (7b) models demonstrate performance comparable to GPT-4. Specifically, the latter’s small size makes it an attractive option to be implemented in RAG pipelines. However, it’s worth noting that none of the open-source models excelled in generating references accurately. Frequently, these models provided incorrect references or failed to generate references altogether. Additionally, we observed substantial improvements in the explanations provided by Llama3.1:8b^80^ compared to those of Llama2^81^. Notably, Llama3.1:8b indicates when citations are hypothetical by stating “generated citations are hypothetical” if the references are inaccurate. This feature significantly reduces the likelihood of misinformation appearing in the explanations generated by Llama3.1:8b. Based on these observations, we conclude that we can be optimistic about using open-source LLMs in place of proprietary models. Note that these open-source explanations can further be improved with other techniques, such as prompt engineering and/or finetuning. We did not investigate this aspect as it is beyond the scope of our study.

**Table 2:** Evaluation of open-source LLM-generated explanations in XpertAI

LLM	Num. parameters and (Ollama quant. level)	Accurately describes each feature and how it is related to the target	Accurately describes how the target can be altered w.r.t. each feature	Lists and explains additional features	Accuracy of generated references	Average RougeL score ± SD
Llama3.1^80^	8B	4	4	5	1.8	0.6 ± 0.02
	(Q4_0)					
Llama2^81^	7B	0	1	2	0.3	0.52 ± 0.05
	(Q4_0)					
mixtral:8 × 7b-instruct-v0.1^82^	8 × 7B	4	5	5	1.25	0.49 ± 0.04
	(Q5_0)					
Phi-2^84^	7B	1	0	3	0	0.38 ± 0.06
	(Q4_0)					
Starling-LM:7b-alpha^83^	7B	5	2	4	1.6	0.46 ± 0.02
	(Q4_0)					
XpertAI (GPT-4o)^30^	N/A	5	5	5	0.82	0.46 ± 0.05

The total scores of all 5 tasks are given here. Highest score = 5. GPT-4o is the default in XpertAI. B stands for Billion in num.parameters column.

## Conclusion and outlook

XAI is becoming increasingly important in ML workflows due to developmental, scientific, and regulatory needs. In this context, we addressed a key challenge in applying XAI to chemistry—the lack of interpretability and scientific grounding of explanations generated by XAI tools. Generally, XAI tools are developed with technical experts in mind, thereby reducing usability. We proposed “XpertAI”, a framework leveraging XAI and LLMs to generate intelligent natural language explanations of structure–property relationships from raw chemistry data. In other words, XpertAI can be perceived as an LLM agent for hypotheses generation that consists of two main components: (1) XAI methods and (2) a RAG model. XpertAI produces readily interpretable and specific explanations while uncovering structure–property relationships. We showed integrating XAI and LLMs is more powerful than using either alone. Furthermore, we demonstrated that this combination can accurately explain input–output relationships, not just model predictions.

We would like to highlight that XpertAI’s performance is limited by (a) the surrogate model’s fit, (b) feature descriptions, and (c) the RAG model’s performance (literature retrieval and augmented text generation). Firstly, if the surrogate model has acquired spurious data relationships, it will inevitably yield an inaccurate explanation. In the current version of XpertAI, hyperparameters are hardcoded to enhance non-expert usability. In our upcoming work, we plan to integrate automated hyperparameter optimization. Additionally, we aim to incorporate other ML models and enable user-provided models, giving more flexibility. However, if a user intends to implement an existing model, they can do so easily using the GitHub codebase.

On the other hand, the quality of the explanations is governed by the descriptions of the feature labels and the RAG model’s performance. For instance, if the features are not found in the literature, the precision of the generated explanations will be low. However, XpertAI is prompted to output “an explicit relationship was not found in the given documents” if it fails to find literature evidence. It is important to note that feature selection significantly impacts the fit of the surrogate model. Therefore, careful selection of input features is crucial within the XpertAI framework. Enhancing the performance and efficiency of the RAG model is an ongoing topic of investigation that is beyond the scope of our work. However, we anticipate that improvements in existing tools and methodologies will enhance XpertAI’s overall performance. For example, better-performing retrievers and LLMs will undoubtedly improve XpertAI’s capabilities. The current version of XpertAI serves as a proof of concept that XAI integrated with LLMs can be a proxy for hypothesis generation in Chemistry. XpertAI mimicks the workflow a scientist would follow to arrive at a hypothesis—following an observation, a hypothesis is generated and supported with literature evidence. Given the growth of tools and methodologies based on LLMs, we aim that XpertAI can become a powerful hypothesis-generating agent in the future.

As we showed previously, open-source LLMs exhibit encouraging signs to be used in place of proprietary GPT models in RAG models. Therefore, our future work will incorporate streamlining the use of open-source LLMs into XpertAI as an alternative to GPT-4 dependencies. This will further increase XpertAI’s accessibility to generate accurate explanations.

Despite current limitations, XpertAI demonstrates potential as an interpretable approach that combines XAI and LLMS for uncovering novel structure–property relationships and generating scientific insights in chemistry. By leveraging AI’s strengths in explanation and language, XpertAI accelerates scientific progress through chemical knowledge extraction and hypothesis generation. This exciting advancement elucidates meaningful chemical structure–property relationships, thereby propelling discovery.

## Supplementary information

Supplementary Information

## Data Availability

Code to XpertAI can be found at https://github.com/geemi725/XpertAI and the XpertAI App can be found at https://xpert-ai.streamlit.app/.

## Data availability

Relevant data can be found at https://github.com/geemi725/XpertAI.
