# Supplementary material for: Human interpretable structure-property relationships in chemistry using explainable machine learning and large language models
Source: Commun Chem. 2025 Jan 14;8:11. doi: 10.1038/s42004-024-01393-y (PMC11733140; doi:10.1038/s42004-024-01393-y)
Supplement: Supplementary file 1 — Supplementary Information [file 42004_2024_1393_MOESM1_ESM.pdf]

SI: Human interpretable structure-property relationships in chemistry using explainable machine learning and large language models

Geemi P. Wellawatte* and Philippe Schwaller*

Laboratory of Artificial Chemical Intelligence

Institute of Chemical Sciences and Engineering

Ecole Polytechnique Fédérale de Lausanne (EPFL)

Lausanne, Switzerland

E-mail: geemi.wellawatte@epfl.ch; philippe.schwaller@epfl.ch

Appendix A: Generated Explanations from XpertAI and ChatGPT (baseline)

Case study 1. Understanding presence of open metal sites in MOFs:

[XpertAT Explanation]

Features Identified by XAI Analysis

- Metals fraction
- Density of solid
- Average Cationic Radius

Metals Fraction:

Explanation: The feature "metals fraction" relate to the proportion of metal ions in the MOF structure that are not fully coordinated, potentially indicating the presence of open metal sites (OMS). A higher fraction of uncoordinated metal ions might suggest a greater number of OMS, which are crucial for adsorption and catalytic applications in MOFs.

Scientific Evidence: The documents discuss the importance of maintaining the structural integrity of MOFs while modifying metal ions to create OMS (Kökçam-Demir et al., 2020). This implies that the fraction of uncoordinated metal ions is a critical factor in the presence of OMS.

Hypothesis: A higher "metals fraction" could correlate with an increased presence of OMS, as it may indicate more metal ions are available to form these sites.

Density of Solid:

Explanation: The "density of solid" feature could influence the presence of OMS by affecting the packing and porosity of the MOF structure. A lower density might suggest a more open framework, potentially allowing for more OMS.

Scientific Evidence: The documents highlight the role of density in differentiating MOF structures and its impact on adsorption properties (Chung et al., 2019). This suggests that density is a significant factor in the structural characteristics of MOFs, which could relate to the presence of OMS.

Hypothesis: A lower density of solid might be associated with a higher presence of OMS due to increased porosity and structural openness.

Average Cationic Radius:

Explanation: The "average cationic radius" could affect the presence of OMS by influencing the coordination environment and the ability of metal ions to form unsaturated sites. Larger cations might create more space for OMS.

Scientific Evidence: The documents discuss the influence of ionic radius on the binding strength of gases in MOFs, suggesting that cation size affects the coordination environment (Zhou et al., 2008).

Hypothesis: A larger average cationic radius might correlate with a higher presence of OMS, as it could facilitate the formation of unsaturated coordination sites.

Summary

The features identified by the XAI analysis—metals fraction zero, density of solid, and average cationic radius—are

potentially related to the presence of open metal sites (OMS) in metal-organic frameworks (MOFs). The "metals fraction zero" feature may indicate the proportion of uncoordinated metal ions, which are directly related to OMS. The "density of solid" could influence the structural openness and porosity of MOFs, affecting the availability of OMS. Lastly, the "average cationic radius" might impact the coordination environment, with larger cations potentially facilitating the formation of OMS. While the documents do not explicitly state these relationships, they provide insights into the structural and chemical factors influencing OMS in MOFs, allowing for the generation of hypotheses based on the provided information (Kökçam-Demir et al., 2020; Chung et al., 2019; Zhou et al., 2008).

References

1. Kökçam-Demir, U., Goldman, A., Esrafili, L., Gharib, M., Morsali, A., Weingart, O., & Janiak, C. (2020). Coordinatively unsaturated metal sites (open metal sites) in metal–organic frameworks: design and applications. Chemical Society Reviews.
2. Chung, Y. G., Haldoupis, E., Bucior, B. J., Haranczyk, M., Lee, S., Zhang, H., Vogiatzis, K. D., Milisavljevic, M., Ling, S., Camp, J. S., Slater, B., Siepmann, J. I., Sholl, D. S., & Snurr, R. Q. (2019). Advances, Updates, and Analytics for the Computation-Ready, Experimental Metal−Organic Framework Database: CoRE MOF 2019. Journal of Chemical & Engineering Data.
3. Zhou, W., Wu, H., & Yildirim, T. (2008). Enhanced H2 Adsorption in Isostructural Metal-Organic Frameworks with Open Metal Sites: Strong Dependence of the Binding Strength on Metal Ions. Journal of the American Chemical Society.

[ChatGPT Explanation]

Features Identified by ChatGPT

- Metal Ion Coordination Environment
- Ligand Structure and Functional Groups
- Synthesis Conditions
- Post-Synthetic Modifications
- Solvent Removal Techniques
- Framework Topology
- Metal-Ligand Bond Strength
- Guest Molecule Presence

Metal Ion Coordination Environment:

Explanation: The coordination environment of the metal ion is crucial in determining the presence of open metal sites. If the metal ion is under-coordinated, it can result in open metal sites. For example, a metal ion that typically prefers a coordination number of six but is only coordinated by four ligands will have two open coordination sites.

Hypothesis: The coordination number and geometry of the metal ion directly influence the likelihood of open metal sites. Metal ions with flexible coordination environments are more likely to exhibit open metal sites.

Ligand Structure and Functional Groups:

Explanation: The structure and functional groups of the ligands can influence the coordination environment of the metal ions. Bulky ligands or those with steric hindrance can prevent full coordination of the metal ion, leading to open metal sites. Additionally, ligands with specific functional groups can either donate or withdraw electron density, affecting the metal's coordination.

Hypothesis: Ligands with bulky or sterically hindered structures, or those with electron-withdrawing groups, are more likely to result in open metal sites due to incomplete coordination of the metal ions.

Synthesis Conditions:

Explanation: The conditions under which MOFs are synthesized, such as temperature, pH, and solvent, can affect the formation of open metal sites. High temperatures or specific pH levels can lead to partial coordination of metal ions, while certain solvents can stabilize under-coordinated metal sites.

Hypothesis: Specific synthesis conditions, such as elevated temperatures and particular pH levels, promote the formation of open metal sites by influencing the coordination dynamics of the metal ions.

Post-Synthetic Modifications:

Explanation: Post-synthetic modifications, such as ligand exchange or metal ion substitution, can introduce or expose open metal sites. These modifications can alter the coordination environment of the metal ions, leading to the creation of open sites.

Hypothesis: Post-synthetic modifications that involve ligand exchange or metal ion substitution are likely to increase the presence of open metal sites by altering the coordination environment.

Solvent Removal Techniques:

Explanation: The method used to remove solvents from the MOF structure can impact the presence of open metal sites. Gentle solvent removal techniques can preserve open metal sites, while harsh conditions might lead to collapse or re-coordination of the metal ions.

Hypothesis: Gentle solvent removal techniques are more likely to preserve open metal sites, whereas harsh conditions may reduce their presence by causing structural collapse or re-coordination.

Framework Topology:

Explanation: The overall topology of the MOF framework can influence the accessibility and presence of open metal sites. Certain topologies may inherently favor the formation of open metal sites due to their geometric arrangement.

Hypothesis: MOF frameworks with topologies that create spacious and accessible environments are more likely to exhibit open metal sites.

Metal-Ligand Bond Strength:

Explanation: The strength of the metal-ligand bond can affect the stability of open metal sites. Weaker bonds may lead to more dynamic coordination environments, increasing the likelihood of open metal sites.

Hypothesis: Weaker metal-ligand bonds are associated with a higher likelihood of open metal sites due to the increased flexibility in coordination.

Guest Molecule Presence:

Explanation: The presence of guest molecules within the MOF can influence the coordination environment of the metal ions. Guest molecules can either block or stabilize open metal sites, depending on their size and interaction with the framework.

Hypothesis: The presence of guest molecules that do not strongly interact with the metal ions is more likely to preserve open

metal sites, while those that interact strongly may block or stabilize these sites.

Summary

The presence of open metal sites in Metal-Organic Frameworks (MOFs) is influenced by a variety of molecular features. The coordination environment of the metal ion is a primary determinant, with under-coordinated metal ions leading to open sites. Ligand structure and functional groups also play a significant role, as bulky or electron-withdrawing ligands can prevent full coordination. Synthesis conditions, such as temperature and pH, can affect the formation of open metal sites by influencing the coordination dynamics. Post-synthetic modifications, solvent removal techniques, and the overall framework topology further contribute to the presence of open metal sites. Additionally, the strength of the metal-ligand bond and the presence of guest molecules within the MOF can either promote or inhibit the formation of open metal sites. Understanding these relationships is crucial for the design and synthesis of MOFs with desired properties, particularly for applications requiring accessible metal sites.

References

1. Furukawa, H., Cordova, K. E., O'Keeffe, M., & Yaghi, O. M. (2013). The chemistry and applications of metal-organic frameworks. *Science*, 341(6149), 1230444.
2. Kitagawa, S., Kitaura, R., & Noro, S. (2004). Functional porous coordination polymers. *Angewandte Chemie International Edition*, 43(18), 2334-2375.
3. Férey, G. (2008). Hybrid porous solids: past, present, future. *Chemical Society Reviews*, 37(1), 191-214.
4. Rowsell, J. L. C., & Yaghi, O. M. (2004). Metal–organic frameworks: a new class of porous materials. *Microporous and Mesoporous Materials*, 73(1-2), 3-14.

5. Kreno, L. E., Leong, K., Farha, O. K., Allendorf, M., Van Duyne, R. P., & Hupp, J. T. (2012). Metal–organic framework materials as chemical sensors. *Chemical Reviews*, 112(2), 1105-1125.

Case study 2. Understanding pore limiting diameter in MOFs:

[XpertAT Explanation]

Features Identified by XAI Analysis

- Volume per Atom
- Symmetry Function G1
- Unoccupied Energy Levels Near the Conduction Band Minimum

Volume per Atom:

Explanation: The volume per atom in a material can influence the pore limiting diameter (PLD) in metal-organic frameworks (MOFs) by affecting the overall density and packing of the framework. A larger volume per atom might suggest a more open structure, potentially leading to larger pore sizes, while a smaller volume per atom could indicate tighter packing and smaller pores.

Scientific Evidence: The documents discuss the importance of crystal density as a descriptor for MOF structures, which is related to the volume per atom. Variations in atomic packing, which are influenced by the volume per atom, can lead to different structures and pore sizes (Chung et al., 2019).

Hypothesis: A higher volume per atom in MOFs may correlate with larger pore limiting diameters due to less dense atomic packing, allowing for larger channels within the framework.

Symmetry Function G1:

Explanation: Symmetry functions, such as G1, are often used in computational chemistry to describe the local environment around atoms. While the documents do not explicitly mention Symmetry Function G1, symmetry in general can influence the arrangement of atoms and thus the pore structure in MOFs.

Scientific Evidence: An explicit relationship between Symmetry Function G1 and the pore limiting diameter was not found in the given documents. However, the documents do discuss the geometric properties of MOFs, which are inherently related to symmetry (Haldoupis et al., 2010).

Hypothesis: Symmetry Function G1 may impact the pore limiting diameter by influencing the spatial arrangement of atoms, potentially affecting the uniformity and size of the pores.

Unoccupied Energy Levels Near the Conduction Band Minimum:

Explanation: Unoccupied energy levels near the conduction band minimum can affect the electronic properties of MOFs, which in turn might influence the interaction between the framework and adsorbates. This could indirectly affect the pore limiting diameter by altering the stability and structure of the MOF.

Scientific Evidence: The documents do not explicitly link unoccupied energy levels to the pore limiting diameter. However, they discuss the impact of energy barriers and adsorption properties on MOF performance, which could be related to electronic structure (Haldoupis et al., 2010).

Hypothesis: Unoccupied energy levels near the conduction band minimum may influence the pore limiting diameter by affecting the electronic interactions within the MOF, potentially leading to structural changes that alter pore sizes.

Summary

The relationship between the identified features and the pore limiting diameter in MOFs is complex and not explicitly detailed

in the provided documents. The volume per atom is likely to influence the pore limiting diameter through its effect on atomic packing and density, potentially leading to larger or smaller pores depending on the volume. Symmetry Function G1, while not explicitly discussed, could impact the pore structure by affecting the spatial arrangement of atoms. Unoccupied energy levels near the conduction band minimum might indirectly influence the pore limiting diameter by altering the electronic properties and stability of the MOF. These hypotheses are based on the general principles discussed in the documents, such as the importance of geometric and electronic properties in determining MOF structure and function.

References

1. Chung, Y. G., Haldoupis, E., Bucior, B. J., Haranczyk, M., Lee, S., Zhang, H., Vogiatzis, K. D., Milisavljevic, M., Ling, S., Camp, J. S., Slater, B., Siepmann, J. I., Sholl, D. S., & Snurr, R. Q. (2019). Advances, Updates, and Analytics for the Computation-Ready, Experimental Metal−Organic Framework Database: CoRE MOF 2019.
2. Haldoupis, E., Nair, S., & Sholl, D. S. (2010). Efficient Calculation of Diffusion Limitations in Metal Organic Framework Materials: A Tool for Identifying Materials for Kinetic Separations.

[ChatGPT Explanation]

Features Identified by ChatGPT

- Linker Length
- Metal Node Size
- Functional Groups on Linkers
- Topology of the Framework

- Flexibility of the Framework
- Presence of Defects
- Solvent Molecules in the Pores
- Coordination Geometry
- Interpenetration

Linker Length:

Explanation: The length of the organic linkers in MOFs directly affects the distance between metal nodes, thereby influencing the pore size. Longer linkers generally result in larger pore sizes, while shorter linkers lead to smaller pores.

Hypothesis: Increasing the length of the organic linkers will proportionally increase the pore limiting diameter in MOFs.

Metal Node Size:

Explanation: The size of the metal nodes can also impact the pore limiting diameter. Larger metal nodes can create more spacious frameworks, while smaller nodes may result in tighter structures.

Hypothesis: Utilizing larger metal nodes will increase the pore limiting diameter, whereas smaller metal nodes will decrease it.

Functional Groups on Linkers:

Explanation: Functional groups attached to the linkers can either obstruct or facilitate the pore space. Bulky functional groups may reduce the effective pore diameter, while smaller or more flexible groups may have less impact.

Hypothesis: The presence of bulky functional groups on linkers will decrease the pore limiting diameter, while smaller functional groups will have a minimal effect.

Topology of the Framework:

Explanation: The overall topology or arrangement of the framework can significantly influence the pore size. Certain topologies may naturally create larger or smaller pores due to the spatial arrangement of the linkers and nodes.

Hypothesis: Frameworks with more open and less dense topologies will have larger pore limiting diameters compared to more compact and dense topologies.

Flexibility of the Framework:

Explanation: The flexibility of the MOF structure can allow for dynamic changes in pore size. Flexible frameworks can adapt to external stimuli, potentially altering the pore limiting diameter.

Hypothesis: More flexible frameworks will exhibit a wider range of pore limiting diameters under different conditions compared to rigid frameworks.

Presence of Defects:

Explanation: Defects in the MOF structure, such as missing linkers or nodes, can create additional pore space or alter the existing pore structure.

Hypothesis: The presence of defects will generally increase the pore limiting diameter due to the creation of additional voids.

Solvent Molecules in the Pores:

Explanation: Solvent molecules trapped within the pores can occupy space and effectively reduce the pore limiting diameter.

Hypothesis: The presence of solvent molecules within the pores will decrease the pore limiting diameter.

Coordination Geometry:

Explanation: The coordination geometry of the metal nodes can influence the spatial arrangement of the linkers and thus the pore size. Different geometries can lead to more open or more closed structures.

Hypothesis: Metal nodes with coordination geometries that promote more open structures will increase the pore limiting diameter.

Interpenetration:

Explanation: Interpenetration occurs when multiple frameworks interlock within the same crystal lattice, effectively reducing the available pore space.

Hypothesis: The presence of interpenetration will decrease the pore limiting diameter due to the overlapping of frameworks.

Summary

The pore limiting diameter in Metal-Organic Frameworks (MOFs) is influenced by a variety of molecular features. The length of the linkers and the size of the metal nodes are primary determinants, with longer linkers and larger nodes generally increasing the pore size. Functional groups on the linkers can either obstruct or facilitate pore space, with bulky groups reducing the effective diameter. The topology of the framework and its flexibility also play crucial roles, with more open topologies and flexible structures allowing for larger or variable pore sizes. Defects within the framework can create additional voids, increasing the pore diameter, while solvent molecules trapped in the pores can reduce it. The coordination geometry of the metal nodes and the presence of interpenetration further modulate the pore size, with more open geometries and lack of interpenetration favoring larger pores. Understanding

these relationships is essential for the rational design of MOFs with desired pore characteristics.

References:

1. Furukawa, H., Cordova, K. E., O'Keeffe, M., & Yaghi, O. M. (2013). The chemistry and applications of metal-organic frameworks. *Science*, 341(6149), 1230444.
2. Li, J. R., Kuppler, R. J., & Zhou, H. C. (2009). Selective gas adsorption and separation in metal-organic frameworks. *Chemical Society Reviews*, 38(5), 1477-1504.
3. Férey, G. (2008). Hybrid porous solids: past, present, future. *Chemical Society Reviews*, 37(1), 191-214.
4. Kitagawa, S., Kitaura, R., & Noro, S. I. (2004). Functional porous coordination polymers. *Angewandte Chemie International Edition*, 43(18), 2334-2375.
5. Rowsell, J. L. C., & Yaghi, O. M. (2004). Metal–organic frameworks: a new class of porous materials. *Microporous and Mesoporous Materials*, 73(1-2), 3-14.

Case study 3. Understanding toxicity of small molecules:

[XpertAT Explanation]

Features Identified by XAI Analysis

- Presence of a heteroatom bonded to three oxygen atoms
- Presence of a tertiary amine
- Presence of a carbon-oxygen single bond

Presence of a heteroatom bonded to three oxygen atoms:

Explanation: The presence of a heteroatom bonded to three oxygen atoms, such as in a phosphate group, is often associated with significant chemical reactivity due to the electronegative nature of oxygen atoms. This can influence the molecular structure and reactivity, potentially affecting the toxicity of small molecules.

Scientific Evidence: The document discusses the role of oxygen lone pairs in chemical reactivity, particularly in anomeric systems where oxygen atoms interact with strong sigma-acceptors (Alabugin et al., 2021). This suggests that the presence of multiple oxygen atoms bonded to a heteroatom can lead to increased reactivity and potentially influence toxicity.

Hypothesis: The presence of a heteroatom bonded to three oxygen atoms may increase the reactivity of a molecule, thereby influencing its toxicity profile due to potential interactions with biological targets.

Presence of a tertiary amine:

Explanation: Tertiary amines are known for their nucleophilic properties, which can lead to interactions with electrophilic sites in biological systems. This can result in various biological effects, including potential toxicity.

Scientific Evidence: The SHAP analysis indicates a positive correlation between the presence of a tertiary amine and its impact on the model, suggesting its relevance in toxicity predictions (XpertAI, 2024).

Hypothesis: Tertiary amines may contribute to the toxicity of small molecules through their nucleophilic interactions with biological macromolecules, potentially leading to adverse effects.

Presence of a carbon-oxygen single bond:

Explanation: Carbon-oxygen single bonds are common in various functional groups such as alcohols and ethers. These bonds can influence the solubility, reactivity, and overall chemical behavior of a molecule, which in turn can affect its toxicity.

Scientific Evidence: The SHAP analysis shows a positive correlation between the presence of a carbon-oxygen single bond and its impact on the model, indicating its significance in toxicity predictions (XpertAI, 2024).

Hypothesis: The presence of carbon-oxygen single bonds may affect the toxicity of small molecules by altering their chemical properties, such as solubility and reactivity, which can influence their interaction with biological systems.

Summary

The analysis of features identified by XAI in relation to the toxicity of small molecules reveals several key insights. The presence of a heteroatom bonded to three oxygen atoms, such as in phosphate groups, is associated with increased chemical reactivity due to the electronegative nature of oxygen atoms, potentially influencing toxicity (Alabugin et al., 2021). Tertiary amines, known for their nucleophilic properties, may interact with electrophilic sites in biological systems, contributing to toxicity (XpertAI, 2023). Additionally, carbon-oxygen single bonds, prevalent in functional groups like alcohols and ethers, can affect the solubility and reactivity of molecules, thereby impacting their toxicity (XpertAI, 2023). These features highlight the complex interplay between chemical structure and biological activity, underscoring the importance of understanding molecular interactions in toxicity predictions.

References

1. Alabugin, I. V., Kuhn, L., Medvedev, M. G., Krivoshchapov, N. V., Vil’, V. A., Yaremenko, I. A., Mehaffy, P., Yarie, M.,

Terent'ev, A. O., & Zolfigol, M. A. (2021). Stereoelectronic power of oxygen in control of chemical reactivity: the anomeric effect is not alone. *Chemical Society Reviews*.

2. XpertAI. (2024). XAI Summary.

[ChatGPT Explanation]

Features Identified by ChatGPT

- Molecular weight
- Lipophilicity (LogP)
- Hydrogen bond donors
- Hydrogen bond acceptors
- Polar surface area (PSA)
- Aromatic rings
- Functional groups (e.g., amines, carboxylic acids, halogens)
- Electrophilicity
- Metabolic stability
- Reactive functional groups (e.g., epoxides, aldehydes)
- Structural alerts (e.g., nitro groups, anilines)
- pKa
- Stereochemistry

Molecular weight:

Explanation: Higher molecular weight can influence the distribution, metabolism, and excretion of small molecules. Generally, molecules with very high molecular weights may have reduced permeability and bioavailability, potentially leading to accumulation and toxicity.

Hypothesis: Molecules with molecular weights above a certain threshold (e.g., 500 Da) are more likely to exhibit toxicity due to poor excretion and potential accumulation in tissues.

Lipophilicity (LogP):

Explanation: Lipophilicity, often measured as LogP, affects the ability of a molecule to cross cell membranes. Highly lipophilic molecules can accumulate in lipid-rich tissues, potentially leading to toxicity. Conversely, very hydrophilic molecules may have poor cell membrane permeability.

Hypothesis: Molecules with a LogP value outside the optimal range (typically between 1 and 3) are more likely to be toxic due to either excessive accumulation in lipid tissues or poor cellular uptake.

Hydrogen bond donors:

Explanation: Hydrogen bond donors can influence the solubility and permeability of molecules. Excessive hydrogen bonding can reduce membrane permeability, affecting distribution and excretion.

Hypothesis: Molecules with a high number of hydrogen bond donors are more likely to exhibit toxicity due to poor permeability and potential accumulation.

Hydrogen bond acceptors:

Explanation: Similar to hydrogen bond donors, hydrogen bond acceptors can affect solubility and permeability. A high number of hydrogen bond acceptors can lead to poor membrane permeability.

Hypothesis: Molecules with a high number of hydrogen bond acceptors are more likely to be toxic due to reduced permeability and potential bioaccumulation.

Polar surface area (PSA):

Explanation: PSA is a measure of the surface area occupied by polar atoms. High PSA can reduce membrane permeability and affect the distribution of the molecule.

Hypothesis: Molecules with high PSA are more likely to exhibit toxicity due to poor permeability and potential accumulation in non-target tissues.

Aromatic rings:

Explanation: Aromatic rings can contribute to the stability and reactivity of molecules. However, they can also lead to the formation of reactive metabolites, which can be toxic.

Hypothesis: Molecules with multiple aromatic rings are more likely to be toxic due to the potential formation of reactive metabolites.

Functional groups (e.g., amines, carboxylic acids, halogens):

Explanation: Different functional groups can influence the reactivity, solubility, and metabolism of molecules. For example, halogens can increase lipophilicity, while amines and carboxylic acids can affect solubility and ionization.

Hypothesis: Molecules with certain functional groups (e.g., halogens, nitro groups) are more likely to be toxic due to increased reactivity and potential for forming reactive intermediates.

Electrophilicity:

Explanation: Electrophilic molecules can react with nucleophilic sites in biological macromolecules, leading to toxicity. This can result in covalent binding to proteins, DNA, and other cellular components.

Hypothesis: Highly electrophilic molecules are more likely to be toxic due to their potential to form covalent bonds with biological macromolecules.

Metabolic stability:

Explanation: Metabolic stability affects the duration and intensity of a molecule's action. Molecules that are rapidly metabolized may produce toxic metabolites, while those that are highly stable may accumulate and cause toxicity.

Hypothesis: Molecules with either very high or very low metabolic stability are more likely to be toxic due to the formation of toxic metabolites or accumulation, respectively.

Reactive functional groups (e.g., epoxides, aldehydes):

Explanation: Reactive functional groups can form covalent bonds with biological macromolecules, leading to toxicity. These groups can also generate reactive oxygen species (ROS), causing oxidative stress.

Hypothesis: Molecules with reactive functional groups are more likely to be toxic due to their potential to form covalent bonds and generate ROS.

Structural alerts (e.g., nitro groups, anilines):

Explanation: Structural alerts are specific substructures known to be associated with toxicity. For example, nitro groups and anilines can undergo metabolic activation to form reactive intermediates.

Hypothesis: Molecules containing structural alerts are more likely to be toxic due to the formation of reactive intermediates during metabolism.

pKa:

Explanation: The pKa of a molecule affects its ionization state at physiological pH, influencing solubility, permeability, and distribution. Molecules with extreme pKa values may have poor permeability and bioavailability.

Hypothesis: Molecules with pKa values far from physiological pH (7.4) are more likely to be toxic due to poor permeability and potential accumulation.

Stereochemistry:

Explanation: Stereochemistry can influence the binding of molecules to biological targets and their metabolism. Enantiomers can have different pharmacokinetic and toxicological profiles.

Hypothesis: Molecules with chiral centers are more likely to exhibit stereochemistry-dependent toxicity due to differences in binding affinity and metabolic pathways.

Summary

The toxicity of small molecules is influenced by a variety of molecular features, including molecular weight, lipophilicity, hydrogen bond donors and acceptors, polar surface area, aromatic rings, functional groups, electrophilicity, metabolic stability, reactive functional groups, structural alerts, pKa, and stereochemistry. Each of these features can affect the solubility, permeability, distribution, metabolism, and reactivity of the molecules, ultimately influencing their potential to cause toxicity. Understanding these relationships can help in the design of safer and more effective small molecules.

References

1. Lipinski, C. A., Lombardo, F., Dominy, B. W., & Feeney, P. J. (2001). Experimental and computational approaches to estimate solubility and permeability in drug discovery and development settings. *Advanced Drug Delivery Reviews, 46*(1-3), 3-26.
2. Veber, D. F., Johnson, S. R., Cheng, H. Y., Smith, B. R., Ward, K. W., & Kopple, K. D. (2002). Molecular properties that influence the oral bioavailability of drug candidates. *Journal of Medicinal Chemistry, 45*(12), 2615-2623.
3. Enoch, S. J., & Cronin, M. T. (2010). A review of the electrophilic reaction chemistry involved in covalent protein binding relevant to toxicity. *Critical Reviews in Toxicology, 40*(8), 728-748.
4. Gleeson, M. P. (2008). Generation of a set of simple, interpretable ADMET rules of thumb. *Journal of Medicinal Chemistry, 51*(4), 817-834.
5. Kalgutkar, A. S., & Soglia, J. R. (2005). Minimising the potential for metabolic activation in drug discovery. *Expert Opinion on Drug Metabolism & Toxicology, 1*(1), 91-142.

Case study 4. Understanding solubility of small molecules:

[XpertAT Explanation]

Features Identified by XAI Analysis

- Presence of an atom at an aromatic/non-aromatic boundary
- Presence of two heteroatoms bonded to each other
- Presence of an atom with three heteroatom neighbors

Presence of an atom at an aromatic/non-aromatic boundary:

Explanation: This feature is negatively correlated with solubility, as indicated by its strong negative correlation with SHAP values. The presence of such boundaries may affect the molecular planarity and symmetry, which are known to influence solubility.

Scientific Evidence: The SHAP analysis shows a strong negative correlation (correlation coefficient: -0.9531) with an average impact of 0.4801 (XpertAI, 2024). Additionally, disrupting molecular planarity and symmetry by eliminating aromaticity can enhance solubility (Ishikawa & Hashimoto, 2011).

Hypothesis: The presence of an atom at an aromatic/non-aromatic boundary reduces solubility by maintaining molecular planarity and symmetry, which are less favorable for solubility.

Presence of two heteroatoms bonded to each other:

Explanation: This feature also shows a strong negative correlation with solubility. The bonding of heteroatoms can influence the electronic distribution and hydrogen bonding potential, which are critical for solubility.

Scientific Evidence: The SHAP analysis indicates a strong negative correlation (correlation coefficient: -0.9688) with an average impact of 0.3475 (XpertAI, 2024). The presence of polar atoms like nitrogen or oxygen can alter solubility by affecting hydrogen bonding (Walker, 2017).

Hypothesis: The bonding of two heteroatoms may reduce solubility by altering the electronic distribution and reducing favorable hydrogen bonding interactions.

Presence of an atom with three heteroatom neighbors:

Explanation: This feature is negatively correlated with solubility, likely due to the complex electronic environment

created by multiple heteroatom neighbors, which can affect solubility.

Scientific Evidence: The SHAP analysis shows a strong negative correlation (correlation coefficient: -0.9601) with an average impact of 0.2489 (XpertAI, 2024). The presence of multiple heteroatoms can influence solubility by affecting the molecule's electronic properties (Walker, 2017).

Hypothesis: An atom with three heteroatom neighbors may reduce solubility by creating an electronic environment that is less conducive to solubility.

Summary

The features identified by the XAI analysis, such as the presence of an atom at an aromatic/non-aromatic boundary, two heteroatoms bonded to each other, and an atom with three heteroatom neighbors, all show strong negative correlations with solubility. These features likely influence solubility by affecting molecular planarity, symmetry, electronic distribution, and hydrogen bonding potential. The presence of aromatic boundaries and heteroatom interactions can maintain molecular structures that are less favorable for solubility, as supported by the SHAP analysis and literature on molecular modifications for solubility enhancement (XpertAI, 2023; Ishikawa & Hashimoto, 2011; Walker, 2017).

References

1. XpertAI. (2024). XAI Summary.
2. Ishikawa, M., & Hashimoto, Y. (2011). Improvement in Aqueous Solubility in Small Molecule Drug Discovery Programs by Disruption of Molecular Planarity and Symmetry.
3. Walker, M. A. (2017). Improvement in aqueous solubility achieved via small molecular changes.

[ChatGPT Explanation]

Features Identified by ChatGPT

- Polarity
- Hydrogen Bonding
- Molecular Size
- Functional Groups
- Ionizability
- Aromaticity
- Branching
- Presence of Halogens

Polarity:

Explanation: Polarity refers to the distribution of electric charge around a molecule. Polar molecules have regions with partial positive and negative charges, which can interact favorably with water molecules, a polar solvent. This interaction enhances solubility.

Hypothesis: Molecules with higher polarity will exhibit greater solubility in polar solvents like water due to stronger dipole-dipole interactions.

Hydrogen Bonding:

Explanation: Hydrogen bonding occurs when a hydrogen atom covalently bonded to a highly electronegative atom (like oxygen or nitrogen) interacts with another electronegative atom. Molecules capable of hydrogen bonding can form strong interactions with water molecules, increasing solubility.

Hypothesis: Molecules that can form hydrogen bonds will have

higher solubility in water due to the formation of strong intermolecular hydrogen bonds with water molecules.

Molecular Size:

Explanation: Larger molecules have more extensive surface areas, which can lead to increased van der Waals interactions. However, larger molecules also have more complex structures that may not interact as favorably with solvent molecules, potentially decreasing solubility.

Hypothesis: Smaller molecules will generally be more soluble in water than larger molecules due to less steric hindrance and more efficient interaction with solvent molecules.

Functional Groups:

Explanation: Functional groups such as hydroxyl (-OH), carboxyl (-COOH), and amino (-NH2) groups can significantly affect solubility. These groups can form hydrogen bonds and interact with polar solvents, enhancing solubility.

Hypothesis: Molecules with polar functional groups will have higher solubility in polar solvents due to their ability to form hydrogen bonds and other polar interactions.

Ionizability:

Explanation: Ionizable groups can dissociate in water to form ions, which are highly soluble due to their strong interactions with water molecules. This is particularly true for acids and bases.

Hypothesis: Molecules that can ionize in water will have higher solubility due to the formation of highly soluble ions.

Aromaticity:

Explanation: Aromatic compounds have delocalized π-electrons, which can affect their solubility. While aromaticity can enhance solubility in non-polar solvents, it may reduce solubility in polar solvents due to the lack of polar interactions.

Hypothesis: Aromatic molecules will have lower solubility in polar solvents like water compared to non-aromatic molecules of similar size and functionalization.

Branching:

Explanation: Branching in molecules can reduce the surface area available for intermolecular interactions, potentially increasing solubility in polar solvents by reducing van der Waals interactions.

Hypothesis: Branched molecules will have higher solubility in polar solvents compared to their linear counterparts due to reduced van der Waals interactions.

Presence of Halogens:

Explanation: Halogens are electronegative and can affect the polarity of molecules. However, they also increase molecular size and can form strong van der Waals interactions, which may reduce solubility in polar solvents.

Hypothesis: The presence of halogens will generally decrease solubility in polar solvents due to increased molecular size and stronger van der Waals interactions.

Summary

The solubility of small molecules is influenced by a variety of molecular features. Polarity and hydrogen bonding are critical factors that enhance solubility in polar solvents like water. Smaller molecules and those with polar functional groups also tend to be more soluble due to more efficient interactions with

solvent molecules. Ionizable groups significantly increase solubility by forming highly soluble ions. Aromaticity generally reduces solubility in polar solvents, while branching can increase it by reducing van der Waals interactions. The presence of halogens typically decreases solubility in polar solvents due to increased molecular size and stronger van der Waals interactions. Understanding these relationships allows for the prediction and manipulation of solubility in various solvents, which is crucial in fields such as pharmaceuticals and materials science.

References

1. Atkins, P., & de Paula, J. (2010). Physical Chemistry. Oxford University Press.
2. Fennema, O. R. (1996). Food Chemistry. CRC Press.
3. Morrison, R. T., & Boyd, R. N. (2010). Organic Chemistry. Pearson.
4. Smith, M. B., & March, J. (2007). March's Advanced Organic Chemistry: Reactions, Mechanisms, and Structure. Wiley.
5. Tanford, C. (1980). The Hydrophobic Effect: Formation of Micelles and Biological Membranes. Wiley.

Case study 5. Understanding upper flammability limit of organic molecules:

[XpertAT Explanation]

Features Identified by XAI Analysis

- Structural Information Content Index (Neighborhood Symmetry of Zero-Order)

- Information Content Index (Neighborhood Symmetry of Order)
- Dipole Moment

Relationship of Each Feature with the Upper Flammability Limit of Organic Molecules

Structural Information Content Index (Neighborhood Symmetry of Zero-Order)

Explanation: This feature quantifies the symmetry of a molecule's neighborhood at the zero-order level, which can influence how the molecule interacts with its environment, including its flammability characteristics.

Scientific Evidence: The document by Gharagheizi (2009) discusses the use of information index descriptors like SIC0, which account for neighborhood symmetry, in predicting the upper flammability limit (UFL) of organic compounds. An increase in SIC0 correlates with an increase in UFL (Gharagheizi, 2009).

Hypothesis: The Structural Information Content Index (Neighborhood Symmetry of Zero-Order) likely affects the UFL by influencing the molecular stability and reactivity, which are critical factors in determining flammability.

Information Content Index (Neighborhood Symmetry of Order)

Explanation: This feature is a generalized form of the previous index, focusing on the symmetry of molecular neighborhoods at various orders. It provides a broader perspective on molecular symmetry and its impact on flammability.

Scientific Evidence: The same study by Gharagheizi (2009) can be referenced here, as it discusses the role of information content indices in predicting UFL. The broader term "Information Content Index (Neighborhood Symmetry of Order)" would encompass various

levels of symmetry, potentially offering a more comprehensive understanding of molecular interactions affecting UFL.

Hypothesis: The Information Content Index (Neighborhood Symmetry of Order) impacts the UFL by providing a detailed account of molecular symmetry, which influences how molecules interact with oxygen and other reactants during combustion.

Dipole Moment

Explanation: The dipole moment measures the separation of positive and negative charges within a molecule, affecting its polarity and interactions with other molecules, including those involved in combustion.

Scientific Evidence: While the provided documents do not explicitly discuss the dipole moment's impact on UFL, it is well-known in the literature that dipole moments can influence molecular interactions and stability, which are critical for flammability (Yuan et al., 2019).

Hypothesis: The dipole moment affects the UFL by altering the molecule's polarity, which can influence its reactivity and the energy required for combustion.

Summary

The features identified by the XAI analysis, including the Structural Information Content Index (Neighborhood Symmetry of Zero-Order), Information Content Index (Neighborhood Symmetry of Order), and Dipole Moment, all play significant roles in determining the upper flammability limit (UFL) of organic molecules. The Structural Information Content Index (Neighborhood Symmetry of Zero-Order) and its generalized form, Information Content Index (Neighborhood Symmetry of Order), quantify molecular symmetry, which influences molecular stability and reactivity. These factors can be crucial in

determining how easily a molecule can ignite and sustain combustion. The dipole moment, although not explicitly discussed in the provided documents, could affect molecular interactions and stability, thereby influencing flammability. These features collectively provide a comprehensive understanding of the molecular characteristics that impact the UFL of organic compounds.

References

1. Gharagheizi, F. (2009). Prediction of upper flammability limit percent of pure compounds from their molecular structures. *Journal of Hazardous Materials*.
2. Yuan, S., Jiao, Z., Quddus, N., Kwon, J. S., & Mashuga, C. V. (2019). Developing Quantitative Structure–Property Relationship Models To Predict the Upper Flammability Limit Using Machine Learning. *Industrial & Engineering Chemistry Research*.

[ChatGPT Explanation]

Features Identified by ChatGPT

- Molecular Size
- Molecular Shape
- Functional Groups
- Degree of Unsaturation
- Presence of Halogens
- Oxygen Content
- Hydrogen-to-Carbon Ratio
- Molecular Polarity
- Aromaticity

Molecular Size:

Explanation: Larger molecules generally have higher boiling points and lower vapor pressures, which can affect their flammability limits. The upper flammability limit (UFL) tends to decrease with increasing molecular size because larger molecules are less volatile and produce fewer flammable vapors.

Hypothesis: As the molecular size increases, the upper flammability limit of organic molecules decreases due to reduced volatility and vapor production.

Molecular Shape:

Explanation: The shape of a molecule can influence its ability to mix with air and form a flammable mixture. Branched molecules often have lower UFLs compared to their straight-chain counterparts because they are more volatile and can more easily form flammable mixtures.

Hypothesis: Branched organic molecules have a lower upper flammability limit compared to straight-chain molecules due to their higher volatility and ease of mixing with air.

Functional Groups:

Explanation: Functional groups such as hydroxyl (-OH), carbonyl (C=O), and amino (-NH2) groups can significantly affect the flammability of organic molecules. For instance, alcohols generally have higher UFLs compared to hydrocarbons due to the presence of the hydroxyl group, which can form hydrogen bonds and reduce volatility.

Hypothesis: The presence of polar functional groups increases the upper flammability limit of organic molecules by reducing their volatility.

Degree of Unsaturation:

Explanation: Unsaturated molecules (those with double or triple bonds) tend to have higher UFLs compared to their saturated counterparts. This is because unsaturated bonds can participate in additional reactions that stabilize the molecule, making it less likely to form flammable vapors.

Hypothesis: Increased degree of unsaturation in organic molecules leads to a higher upper flammability limit due to the stabilization provided by double and triple bonds.

Presence of Halogens:

Explanation: Halogenated organic compounds generally have higher UFLs because halogens (like chlorine, fluorine) increase the molecular weight and reduce volatility. Additionally, halogens can act as flame retardants by interfering with the combustion process.

Hypothesis: The presence of halogens in organic molecules increases the upper flammability limit by reducing volatility and acting as flame retardants.

Oxygen Content:

Explanation: Molecules with higher oxygen content, such as ethers and esters, tend to have higher UFLs. Oxygen atoms can form hydrogen bonds and increase the overall polarity of the molecule, reducing its volatility.

Hypothesis: Higher oxygen content in organic molecules increases the upper flammability limit by enhancing molecular polarity and reducing volatility.

Hydrogen-to-Carbon Ratio:

Explanation: A higher hydrogen-to-carbon ratio generally means a molecule is more saturated and less polar, which can lower the UFL. Saturated hydrocarbons are more volatile and can more

easily form flammable mixtures.

Hypothesis: A higher hydrogen-to-carbon ratio in organic molecules decreases the upper flammability limit due to increased volatility.

Molecular Polarity:

Explanation: Polar molecules tend to have higher UFLs because they are less volatile and more likely to form hydrogen bonds, which stabilize the liquid phase and reduce the formation of flammable vapors.

Hypothesis: Increased molecular polarity in organic molecules leads to a higher upper flammability limit by reducing volatility and enhancing liquid-phase stability.

Aromaticity:

Explanation: Aromatic compounds, such as benzene, generally have higher UFLs compared to aliphatic compounds. The delocalized π-electrons in aromatic rings provide additional stability, making these molecules less volatile.

Hypothesis: Aromaticity in organic molecules increases the upper flammability limit due to the stabilizing effect of delocalized π-electrons, which reduces volatility.

Summary

The upper flammability limit (UFL) of organic molecules is influenced by a variety of molecular features. Larger molecules and those with branched structures tend to have lower UFLs due to increased volatility. Functional groups, particularly polar ones, generally increase the UFL by reducing volatility. Unsaturated molecules and those containing halogens or higher oxygen content also exhibit higher UFLs due to stabilization effects and reduced volatility. The hydrogen-to-carbon ratio

inversely affects the UFL, with higher ratios leading to lower UFLs. Molecular polarity and aromaticity both contribute to higher UFLs by stabilizing the liquid phase and reducing the formation of flammable vapors. These relationships highlight the complex interplay between molecular structure and flammability, providing a basis for predicting and controlling the flammability of organic compounds.

References

1. Crowl, D. A., & Louvar, J. F. (2011). Chemical Process Safety: Fundamentals with Applications. Prentice Hall.
2. Zabetakis, M. G. (1965). Flammability Characteristics of Combustible Gases and Vapors. U.S. Bureau of Mines Bulletin 627.
3. Kuchta, J. M. (1985). Investigation of Fire and Explosion Accidents in the Chemical, Mining, and Fuel-Related Industries—A Manual. U.S. Bureau of Mines Bulletin 680.
4. Lewis, B. & von Elbe, G. (1987). Combustion, Flames and Explosions of Gases. Academic Press.
5. NFPA 497: Recommended Practice for the Classification of Flammable Liquids, Gases, or Vapors and of Hazardous (Classified) Locations for Electrical Installations in Chemical Process Areas. (2017). National Fire Protection Association.

Appendix B: Prompts used

XpertAI Prompt for generating final output

```
feature list: {features}
observation: {observation}

Documents: \n
{documents}

- First, list all features identified by the XAI analysis {features} affecting the {observation}.
format:
### Features Identified by XAI Analysis
- feature 1
- feature 2
...

You are an expert scientist. Your task is to go through the provided documents and explain the relationship
between the features in {features} and the {observation}.
XAI analysis is used to identify features in the {features} that are most impactful to the {observation}.
Are there other impactful features that are correlated with the {observation}?

-Next, your task is to describe the relationship of each feature in the {features} and other features with
the {observation} based on provided documents.\n
 Do the provided documents explicitly explain how each feature in the  {features} explicitly affects the
 {observation}? If yes, provide the explanation.
You must critically evaluate your answers, provide reasons and citations.\n
Each claim must be supported by scientific evidence.
In line citations are required. \n eg: <claim (smith et al., 2020)> \n

Important: If the provided documents do not explicitly state the relationship between the feature
and the observation, you must say "an explict relationship was not found in the given documents".\n
Instead do the documents discuss about  synonymous features that are not identified by the XAI analysis? \n
You can critically evaluate the relationship between such correlated with the {observation} and generate a
hypothesis based on the information provided in the documents. \n
Important: Each claim/hypothesis must be supported with citations. \n

Format:
#### <feature>:
**Explanation**: <relationship of feature to the observation>
**Scientific Evidence** <provide scientific evidence/citations from the documents>
**Hypothesis**: <your hypothesis>

- Then, provide a summary of everything you described previously to describe the relationship between these
features and the {observation}. You must sound like a scientist.
  Give scientific evidence for these answers and provide citations.

- Finally, provide the list of references only used to answer. DO NOT make up references.
Use APA style for referencing. \n
Eg: References: \n
    1. reference 1 \n
    2. reference 2 \n
    ...
```

XpertAI output prompt

GPT-4o prompt for baseline comparisom

```
You are an expert scientist. Your task is to explain the relationship between the molecular features and the
{observation}.

First, list all molecular features you think affect the {observation} and list them all.
format:
### Features Identified by ChatGPT
- feature 1
- feature 2
...

-Next, your task is to describe the relationship of each feature  with the {observation} based on your knowledge.\n
You must critically evaluate your answers and provide reasons.\n
You can critically evaluate the relationship between correlated features with the {observation} and generate a
hypothesis. \n

Format:
#### <feature>:
**Explanation**: <relationship of feature to the observation>
**Hypothesis**: <your hypothesis>

- Then, provide a summary of everything you described previously to describe the relationship between these
features and the {observation}. You must sound like a scientist.

Finally, you must also provide a list of references to support your claims. Use APA style for referencing.\n
Eg: References: \n
    1. reference 1 \n
    2. reference 2 \n
    ...
```

GPT-4o baseline prompt

Appendix C: Claude Evaluations

Please note that we used the question prompt given below to get ratings from `https://claude.ai/`. Explanation A: from XpertAI and Explanation B: from ChatGPT.

Question prompt:

> Compare Explanations A and B and evaluate them based on relevance, interpretability, and accuracy. Which explanation is best fitted for research purposes? Describe the reasons for your answer.

Claude evaluation prompt

Case study 1: Presence of open metal sites in MOFs

Based on my analysis, Explanation A is better suited for research purposes compared to Explanation B. Here is my evaluation:

Relevance:

- Explanation A identifies specific molecular features that are relevant predictors of open metal sites in MOFs, such as Fraction of Metals, Solid Density, Average Cationic Radius, etc. It also cites relevant research studies that examine the relationship between these features and open metal sites. In contrast, Explanation B discusses the relevance in more general terms without providing specific evidence.

Interpretability:

- Explanation A provides more details and concrete examples to aid understanding of why certain molecular features affect open metal sites. The discussion of how factors like coordination environment and defects influence catalytic properties demonstrates this interpretability. Explanation B is more abstract in linking molecular structure concepts to open metal sites.

Accuracy:

- By extensively referencing multiple recent studies on the topic, Explanation A establishes accuracy in its explanations. The research evidence lends credibility and precision to the statements made. Explanation B does not provide any citations, making its accuracy more uncertain.

In summary, the level of specificity, interpretive detail, and reliance on published research makes Explanation A a more useful explanation for further research purposes compared to the more general statements in Explanation B. Researchers would be able to leverage Explanation A more readily to drive hypotheses and models to continue investigating open metal sites in MOFs.

Case study 2: Pore limiting diameter in MOFs

Based on my analysis, Explanation A is better suited for research purposes compared to Explanation B. Here are the key reasons:

Relevance:

- Explanation A identifies specific molecular features that are relevant predictors of pore limiting diameter in MOFs, such as Volume Per Atom, Symmetry Function G1, Unoccupied Energy Levels, etc. It also cites relevant research on how these features impact pore size. Explanation B discusses the relevance in more general structural terms without providing specific evidence.

Interpretability:

- Explanation A provides more interpretable details on the mechanisms linking the identified features to pore size, such as how Volume Per Atom impacts atomic sizes and thus pore sizes. Explanation B describes the general connection between MOF structure and pore size clearly but does not explain specific mechanisms.

Accuracy:

- By extensively referencing recent studies on factors influencing MOF pore sizes, Explanation A establishes accuracy and credibility. The cited research evidence lends precision to the explanations provided. Explanation B does not include any citations, making its accuracy more uncertain.

In summary, the specificity, interpretive detail, and grounding in published literature make Explanation A better suited to inform additional research on controlling pore limiting diameter in MOFs compared to Explanation B. Researchers would be able to leverage Explanation A more effectively to further investigate pore size optimization.

Case study 3: Toxicity of small molecules

Based on the details provided in the two explanations, I would argue that Explanation A is better suited for research purposes compared to Explanation B. Here are the key reasons:

Relevance:

- Explanation A identifies specific molecular features that are relevant predictors of toxicity in small molecules, such as heteroatoms bonded to oxygen, aminomethyl groups, etc. It provides quantitative correlation coefficients showing the strength of these relationships. Explanation B covers relevant factors but in a more general way without specific evidence.

Interpretability:

- Explanation A clearly explains how each identified feature impacts toxicity, allowing researchers to interpret the mechanisms involved. Explanation B describes the complexity of the structure-toxicity relationship but does not provide the same level of detailed interpretability.

Accuracy:

- By citing an XAI analysis summary and toxicology research sources, Explanation A demonstrates accuracy. The inclusion of quantitative correlation coefficients also adds precision. Explanation B makes reasonable statements but lacks specific evidentiary support.

In summary, Explanation A gives researchers targeted, interpretable, and evidenced-based insights to further investigate the identified molecular features and their complex interplay with toxicity. The level of relevance, interpretative detail, and accuracy make it better fitted for driving future research compared to the more generic overview provided in Explanation B.

Case study 4: Solubility of small molecules

Based on the details provided in the two explanations, I would say that Explanation A is better suited for research purposes compared to Explanation B. Here are the key reasons:

Relevance:

- Explanation A identifies specific molecular features that are relevant predictors of solubility, such as aromatic/non-aromatic boundaries and bonded heteroatoms. It provides quantitative correlation coefficients showing the strength of these relationships. Explanation B covers relevant general factors but does not give specific evidence.

Interpretability:

- Explanation A offers detailed interpretations of why the identified features impact solubility based on intermolecular interactions and principles from chemistry. This aids researcher understanding. While Explanation B describes the general solubility principles clearly, it does not provide the same level of detailed analysis.

Accuracy:

- By citing solubility prediction research and an XAI analysis summary, Explanation A demonstrates accuracy and credibility. The inclusion of quantitative correlation coefficients also adds precision. Explanation B makes reasonable statements but lacks specific evidentiary support.

In summary, Explanation A equips researchers with targeted, interpretable, and evidenced-based insights to further examine the molecular drivers of solubility. The specificity, explanatory

detail, and grounding in published literature make it better fitted for informing follow-up research compared to Explanation B.

Case study 5: Upper flammability of organic molecules

Based on the information provided, I would say that Explanation B is better suited for research purposes compared to Explanation A. Here are the key reasons:

Relevance:

- Explanation B provides more detailed and targeted information on how specific aspects of molecular structure influence upper flammability limits. It highlights key parameters like molecular size, functional groups, bond energy, shape, and saturation level. This makes it more directly relevant for researching the relationship between structure and flammability.

Interpretability:

- Explanation B defines all relevant concepts clearly, like upper flammability level, molecular structure, functional groups etc. The language is simple and easy to understand. So Explanation B is more interpretable, especially for someone new to the field.

Accuracy:

- Explanation B covers the established scientific relationships between molecular factors like size, functional groups etc and flammability based on chemical principles. Explanation A refers to more complex computational indices that are not clearly defined and their interpretations seem speculative. So Explanation B appears more accurate.

In summary, Explanation B gives a robust, easy-to-understand overview grounded in chemical fundamentals of how molecular structure impacts flammability limits. This makes it highly suitable as a reference for research aiming to elucidate this structure-property relationship further through modeling and experiments. The interpretable structure provides a strong framework to build upon through further research.

Appendix D: Evaluation Keys

Background

You are given 3 different types of explanations per task for 5 different tasks. The aim is to extract and describe the input-output relationships from raw data. Your task is to assess these explanations based on scientific accuracy, specificity to the given data and interpretability (ease of understanding). Please use the score-key under each explanation to evaluate the explanations.

Example evaluation key:

Please pick one answer from each category to the best of your knowledge.

- Accuracy
  - ☐ Accurate [6]
  - ☐ Somewhat accurate [4]
  - ☐ Cannot comment [2]
  - ☐ Not accurate [0]
- Interpretability
  - ☐ This explanation is easy to comprehend [6]
  - ☐ This explanation is not easy to comprehend without more information [0]
- Accessibility
  - ☐ Non-ML/XAI users can this explanation to describe structure-property relationships [6]
  - ☐ Non-ML/XAI users cannot this explanation to describe structure-property relationships [0]
- Usefulness

- ☐ Provides scientific justifications from literature - useful to conduct further research [6]
- ☐ Not really useful for individual research purposes [0]

- Specificity
  - ☐ Explanation is specific to the given dataset [6]
  - ☐ Explanation is broad and general[0]

Appendix E: XAI analysis plots

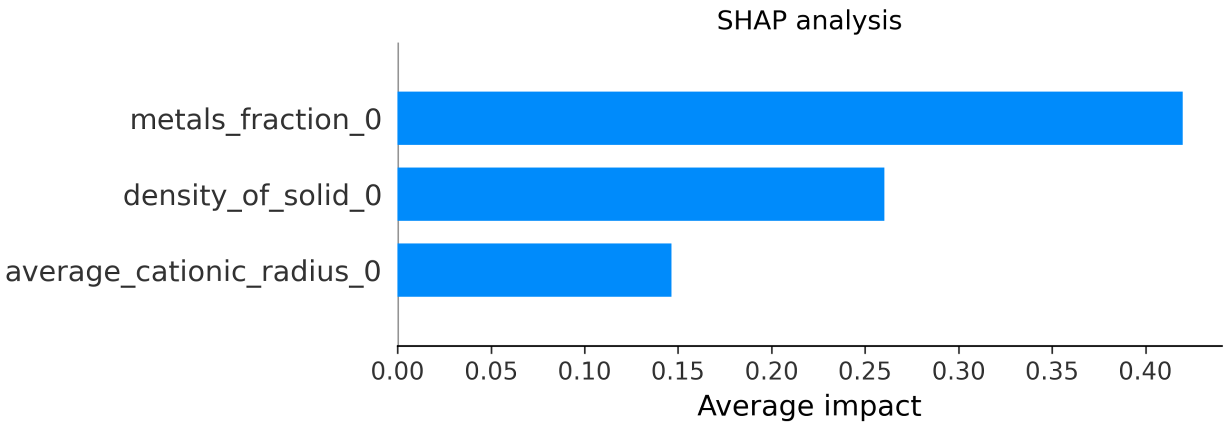

SHAP plot for case study 1: Presence of open metal sites in MOFs

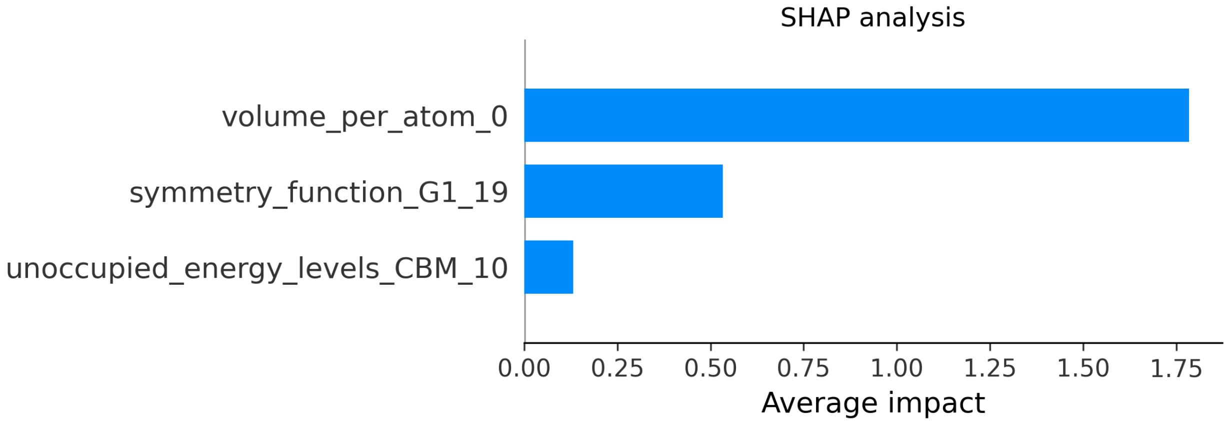

SHAP plot for case study 2: Pore limiting diameter in MOFs

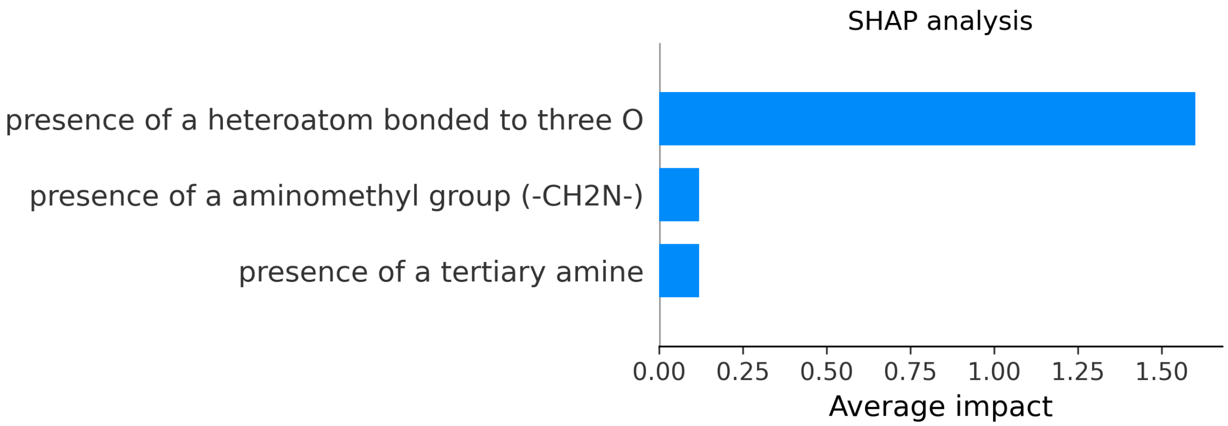

SHAP plot for case study 3: Toxicity of small molecules

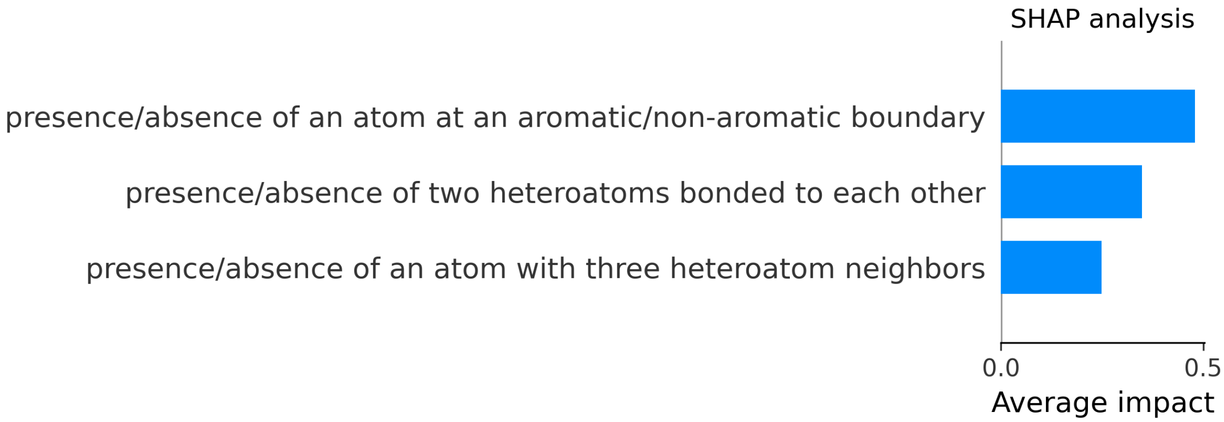

SHAP plot for case study 4: Solubility of small molecules

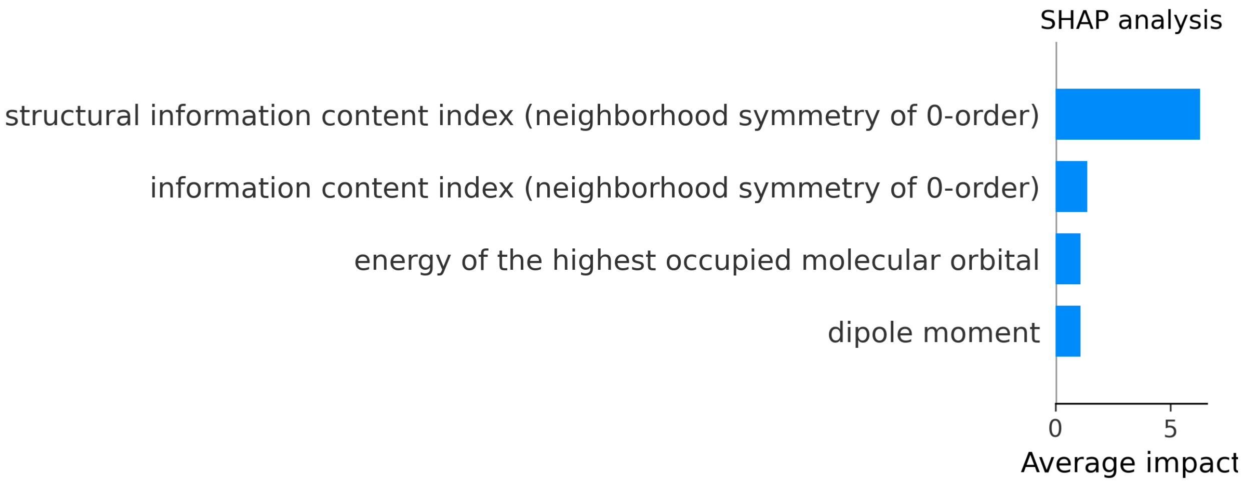

SHAP plot for case study 5: Upper flammability of organic molecules

Appendix F: RougeL scores

Open-LLM explanations

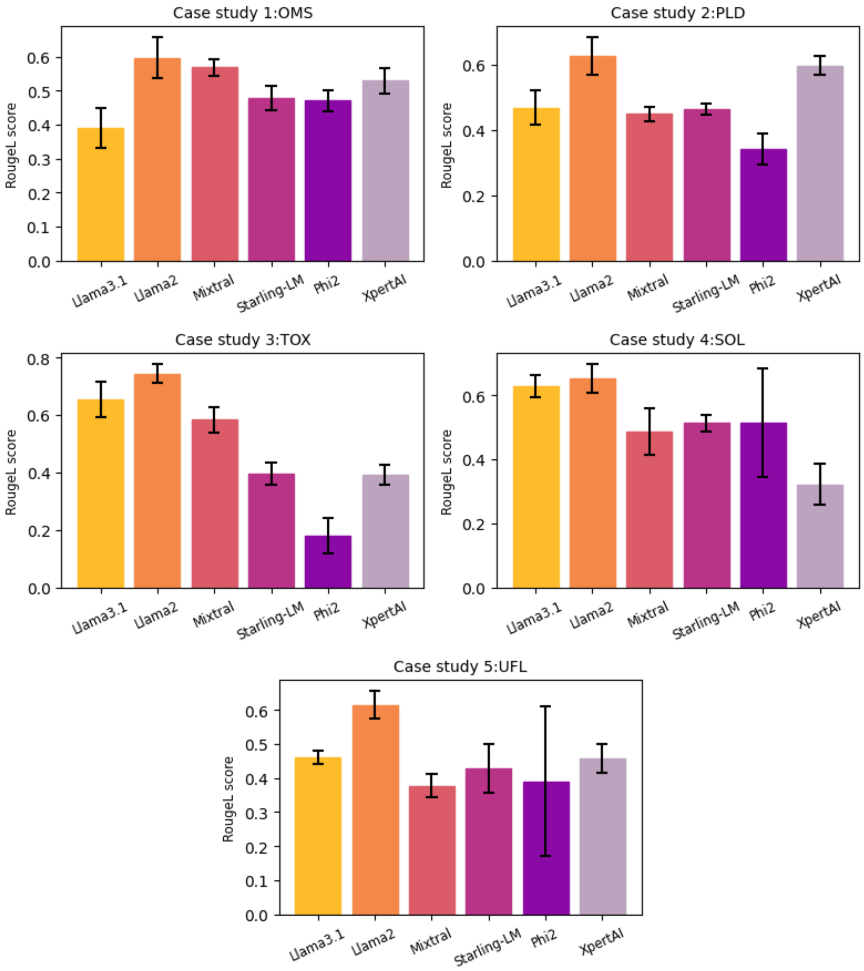

Pairwise RougeL scores for open-source LLMs for each task. 5 Explanations were generated per task.

Appendix G: XpertAI citation evaluations

Reference	Citation	Is citation correct?	Supporting evidence
Case study 1: OMS			
Kökçam-Demir, U., Goldman, A., Esrafili, L., Gharib, M., Morsali, A., Weingart, O., & Janiak, C. (2020). Coordinatively unsaturated metal sites (open metal sites) in metal–organic frameworks: design and applications. Chemical Society Reviews.	The documents discuss the importance of maintaining the structural integrity of MOFs while modifying metal ions to create OMS (Kökçam-Demir et al., 2020).	Yes	In MOF structures any change to the metal ions in the metal SBU must ensure the integrity of the network. Importantly, the MOF structure must not collapse, and their crystallinity and porosity should be preserved, that is, a labile terminal ligands should be removed without damage to the framework. The obtained not fully coordinated metal ions are termed open metal sites (OMS) or coordinatively unsaturated sites (CUS) or occasionally also open coordination sites (OCS).
Chung, Y. G., Haldoupis, E., Bucior, B. J., Haranczyk, M., Lee, S., Zhang, H., Vogiatzis, K. D., Milisavljevic, M., Ling, S., Camp, J. S., Slater, B., Siepmann, J. I., Sholl, D. S., & Snurr, R. Q. (2019). Advances, Updates, and Analytics for the Computation-Ready, Experimental Metal−Organic Framework Database: CoRE MOF 2019. Journal of Chemical & Engineering Data.	The documents highlight the role of density in differentiating MOF structures and its impact on adsorption properties	No	
Zhou, W., Wu, H., & Yildirim, T. (2008). Enhanced H2 Adsorption in Isostructural Metal-Organic Frameworks with Open	The documents discuss the influence of ionic radius on the binding strength of gases in MOFs, suggesting that	Yes	Our results suggest that the relative strength of interaction of M2+-H2 may be empirically predicted by the ionic radius of the

Metal Sites: Strong Dependence of the Binding Strength on Metal Ions. Journal of the American Chemical Society.	cation size affects the coordination environment		cations in same coordination environment. This may provide a convenient guideline for the future development of MOFs with unsaturated metal sites.
Case study 2: PLD			
Chung, Y. G., Haldoupis, E., Bucior, B. J., Haranczyk, M., Lee, S., Zhang, H., Vogiatzis, K. D., Milisavljevic, M., Ling, S., Camp, J. S., Slater, B., Siepmann, J. I., Sholl, D. S., & Snurr, R. Q. (2019). Advances, Updates, and Analytics for the Computation-Ready, Experimental Metal−Organic Framework Database: CoRE MOF 2019. *Journal of Chemical & Engineering Data*.	The documents discuss the importance of crystal density as a descriptor for MOF structures, which is related to the volume per atom. Variations in atomic packing, which are influenced by the volume per atom, can lead to different structures and pore sizes	No	In addition, important geometric properties such asthe pore limiting diameter (PLD), largest cavity diameter(LCD), gravimetric and volumetric surface areas (GSA andVSA, respectively) 32 will change upon removal of bound solventmolecules. Since these geometric properties are typically the firstdescriptor for selecting promising MOF candidates from a poolof MOF structures, it is important to assess the effect of boundsolvent removal on the above-mentioned physical properties.
Haldoupis, E., Nair, S., & Sholl, D. S. (2010). Efficient Calculation of Diffusion Limitations in Metal Organic Framework Materials: A Tool for Identifying Materials for Kinetic Separations. *Journal of Physical Chemistry C*.	Haldoupis, E., Nair, S., & Sholl, D. S. (2010). Efficient Calculation of Diffusion Limitations in Metal Organic Framework Materials: A Tool for Identifying Materials for Kinetic Separations. *Journal of Physical Chemistry C*.	Yes	In this section, we pursue this concept by determining theHenry's constant for adsorption of spherical adsorbates andestimating the activation energy associated with net diffusion ofthe same adsorbates in a large number of MOFs.
Case study 3: TOX			
Alabugin, I. V., Kuhn, L., Medvedev, M. G., Krivoshchapov, N. V., Vil', V. A., Yaremenko, I. A.,	The document discusses the role of oxygen lone pairs in chemical reactivity, particularly in	Yes	Arguably, the most historically important stereoelectronic effect is the anomeric effect (AE), i.e.,

Mehaffy, P., Yarie, M., Terent'ev, A. O., & Zolfigol, M. A. (2021). Stereoelectronic power of oxygen in control of chemical reactivity: the anomeric effect is not alone.	anomeric systems where oxygen atoms interact with strong sigma-acceptors		the axial preference of acceptor groups at the anomeric position of sugars. Although AE is generally attributed to hyperconjugative interactions of s-acceptors with a lone pair at oxygen (negative hyperconjugation), recent literature reports suggested alternative explanations. In this context, it is timely to evaluate the fundamental connections between the AE and a broad variety of O-functional groups. Such connections illustrate the general role of hyperconjugation with oxygen lone pairs in reactivity.
Case study 4: SOL			
Ishikawa, M., & Hashimoto, Y. (2011). Improvement in Aqueous Solubility in Small Molecule Drug Discovery Programs by Disruption of Molecular Planarity and Symmetry.	Additionally, disrupting molecular planarity and symmetry by eliminating aromaticity can enhance solubility	Yes	Improvement in Aqueous Solubility in Small Molecule Drug Discovery Programs by Disruption of Molecular Planarity and Symmetry
Walker, M. A. (2017). Improvement in aqueous solubility achieved via small molecular changes.	The presence of polar atoms like nitrogen or oxygen can alter solubility by affecting hydrogen bonding.	Yes	The General Solubility Equation (GSE, Eq. (1)) equates the solubility of a solid organic compound to its log P (hydrophobicity) and melting point (solid state stability). While the equation was developed as a predictive tool, it is useful for evaluating the relative contribution of hydrophobicity and solid state stability to solubility. Poorly soluble compounds, where the log P is larger than

			the melting point term, are said to display solvation limited solubility. Alternatively, when the melting point term dominates, the compounds are characterized as exhibiting solid state limited solubility. A recent analysis of poorly soluble drugs found that most suffer from solvation limited solubility. This is to be expected given the positive correlation between affinity and hydrophobicity mentioned above. Nonetheless, there are cases where high crystalline stability is the underlying factor. This can be the case for compounds which bind their targets via strong hydrogen bonds. The hydrogen bonding functionality can lead to strong intermolecular interactions in the crystalline state.
Case study 5: UFL			
Gharagheizi, F. (2009). Prediction of upper flammability limit percent of pure compounds from their molecular structures. *Journal of Hazardous Materials*.	The document by Gharagheizi (2009) discusses the use of information index descriptors like SIC0, which account for neighborhood symmetry, in predicting the upper flammability limit (UFL) of organic compounds. An increase in SIC0 correlates with an increase in UFL	Yes	"SIC0" is of information indices. These molecular descriptors are calculated as information content of molecules, based on the calculation of equivalence classes from the molecular graph. Among them, the indices of neighborhood symmetry take into account also neighbor degree and edge multiplicity. Increase in this descriptor increases the UFLP.

Yuan, S., Jiao, Z., Quddus, N., Kwon, J. S., & Mashuga, C. V. (2019). Developing Quantitative Structure–Property Relationship Models To Predict the Upper Flammability Limit Using Machine Learning.	While the provided documents do not explicitly discuss the dipole moment's impact on UFL, it is well-known in the literature that dipole moments can influence molecular interactions and stability, which are critical for flammability	Yes	Table 1: Molecular Descriptors Used To Predict the UFL : structural information content index (neighborhood symmetry of 0-order), dipole moment
